# MYBL2 impedes cisplatin sensitivity through suppressing GSDME-mediated pyroptosis in lung adenocarcinoma.

**DOI:** 10.1038/s41420-026-03175-y

**Published:** 2026-06-02

**Authors:** Tingting Lu, Jie Zhang, Wendi Xuzhang, Xiru Quan, Guangling Jie, Ziming Li, Shun Lu

**Affiliations:** 1 Shanghai Lung Cancer Center, Shanghai Chest Hospital, Shanghai Jiao Tong University School of Medicine, Shanghai, China; 2 State Key Laboratory of Oncology in South China, Collaborative Innovation Centre for Cancer Medicine, Guangdong Esophageal Cancer Institute, Guangzhou, China; Department of Radiation Oncology, Sun Yat-sen University Cancer Center, Guangzhou, China

**Keywords:** Cancer therapy, Molecular biology

## Abstract

The underlying relationship between pyroptosis and tumorigenesis has garnered increasing attention recently. It has been demonstrated that the transcription factor MYB proto-oncogene-like 2 (MYBL2) is elevated in various solid tumors, thereby promoting cell proliferation. However, the role of MYBL2 gene in lung adenocarcinoma (LUAD), especially its potential connection with pyroptosis, is still unclear. Here, by combining ATAC seq and RNA seq methods, we initially identified MYBL2 gene as the main regulatory transcription factor (MR-TF) driving LUAD phenotype plasticity. An in-depth analysis of the sequencing results revealed that the expression of the MYBL2 gene was significantly higher in LUAD tissues than in normal tissues. Further experiments confirmed that MYBL2 promoted the proliferation of LUAD and attenuated tumor cell pyroptosis. Mechanistically, MYBL2 negatively regulated GSDME expression by binding to its promoter, thereby diminishing cisplatin-induced pyroptosis and ultimately leading to reduced chemosensitivity. Moreover, MYBL2 interacted with YAP1 to co-regulate GSDME transcription. The results of this research could be used to inform new strategies for screening effective populations and overcoming treatment resistance, making them potentially clinically valuable.

## Introduction

Lung cancer (LC) is a malignant tumor with the second most commonly diagnosed cancer and the chief cause of cancer death in 2020, accounting for 11.4% of all cancers worldwide [1]. There are about 2.2 million new cases of lung cancer, resulting in 1.6 million deaths globally[1]. Lung cancer is estimated to become the most common type of cancer in China, and LUAD makes up for approximately 70–80% among all cases of lung cancer [2]. Despite advances in diagnostic and therapeutic technologies, the overall survival rate for lung cancer remains relatively low. Pathologically, as reported by the 2015 World Health Organization (WHO) guidelines, LUAD is generally considered to follow a stepwise progression from adenocarcinoma in situ (AIS) or minimally invasive adenocarcinoma (MIA) to invasive adenocarcinoma (IAC). Both AIS and MIA are categorized as the pre-invasive stage of LUAD, while invasive adenocarcinoma (IAC) stands for the invasive stage. Currently, chemotherapy remains one of the standard treatment methods for lung cancer. Cisplatin is a well-established and widely used chemotherapy drug in LUAD treatment. However, there is still a portion of the population that cannot benefit from traditional chemotherapy, suggesting the limitations of its clinical application, so in-depth study of the resistance mechanism becomes urgent and necessary.

Pyroptosis, characterized by cell swelling and membrane rupture, is a newly discovered pattern of programmed cell death and possesses great advantages in many diseases [3, 4]. Pyroptosis is generally induced by activation of gasdermin family proteins composed of gasdermin A, B, C, D, E, and DFNB59 [4–7]. Every gasdermin protein includes a cytotoxic N-terminal domain and a C-terminal regulatory domain, which are linked by a flexible component. When the connection between the two structure domains is hydrolyzed, gasdermin-N domains bind membrane lipids and punch holes, leading to extracellular material infiltration, cell swelling, and ultimately pyroptosis [8]. Emerging findings have demonstrated that various stimuli triggered the activation of gasdermin proteins through distinct pathways, triggering pyroptotic process and releasing related inflammatory cytokines, such as LDH, HMGB1, and cytokines like interleukin-1β and interleukin-18 [9]. Among the gasdmin executors, GSDMD and GSDME (encoded by DFNA5) are typical molecules that are cleaved by caspase, thus resulting in pyroptotic phenomenon [10–14]. Di et al. reported that OTUD4 enhances the radiosensitivity of nasopharyngeal carcinoma by regulating GSDME deubiquitination and affecting pyroptosis [12]. Xin et al. find that ALKBH4 affects 5-FU chemotherapy sensitivity by modulating the H3K4me3 modification of GSDME promoter region [13]. Multiple research studies have elicited a strong correlation between GSDME and tumor chemotherapy [15]. Thus, the induction of tumor cell pyroptosis is still a key issue in current antitumor strategies [16].

The MYB transcription factor family, which is crucial for regulating important cellular biological processes, includes three classical members: MYB (C-MYB), MYBL1 (A-MYB), and MYBL2 (B-MYB) [17]. The primary functions of MYBL2 consist of regulating cell proliferation, determining cell fate, and controlling the activity of cyclin-dependent kinases (CDKs) [18–22]. MYBL2 promotes G1/S transition and enhances G2/M transition by modulating CDKs-related genes [23]. MYBL2 in various tumors is significantly elevated compared to adjacent tissues, which facilitates tumor occurrence, drug resistance, and tumor metastasis [24–27]. In bladder cancer, MYBL2 interacts with FOXM1 promotes proliferation and metastasis through transactivation of CDCA3 [18]. Notch3 regulates MYBL2 to restrict tumor initiation and progression in breast cancer [28]. In prostate cancer, MYBL2 drives tumor plasticity by inhibiting its transcriptional target gene CDK2 [21]. However, the role of MYBL2 in lung adenocarcinoma, especially its potential relationship with pyroptosis, remains to be elucidated.

Here, we combined Assay for Transposase Accessible Chromatin with high-throughput sequencing (ATAC-seq) and RNA-seq on LUAD tumor tissues after surgical resection to investigate the transcriptional profiles. The bioinformatics results showed that the transcription factor MYBL2 expression was significantly elevated in LUAD compared to the adjacent normal tissues. In vitro combined with in vivo experiments confirmed MYBL2 promoted proliferation of LUAD and inhibited tumor pyroptosis. Mechanistically, MYBL2 negatively transactivated GSDME by directly binding to its promoter, thereby diminishing cisplatin-induced pyroptosis and ultimately leading to reduced chemosensitivity. Furthermore, MYBL2 interacted with YAP1 to co-regulate GSDME expression. The results of this research have potential clinical translational value as a scientific basis for new strategies for subsequent screening of effective populations and overcoming treatment resistance.

## Results

### MYBL2 is elevated in LUAD and correlated with shorter progression free survival (PFS)

**Fig. 1:**
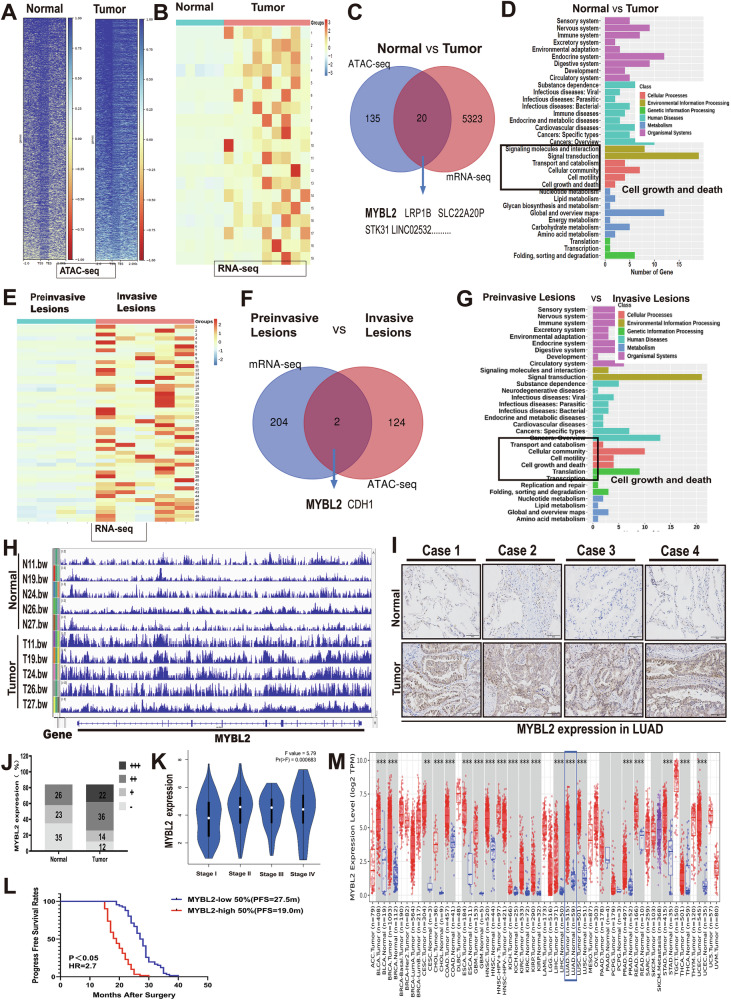
MYBL2 is upregulated and correlated with poor PFS in LUAD. **A** ATAC-seq signals in each sample were concentrated in the transcription start site (TSS) and promoter region −2kb to 2 kb. Sample library preparation was successful. **B** Heat map of differentially expressed genes from RNA-seq of LUAD and normal tissue. **C** The intersection of genes differentiated by ATAC-seq and RNA-seq was identified between tumors and normal tissues. **D** KEGG analysis of peak differential genes in ATAC-seq showed the differential genes mainly involved in cell proliferation and cell death progress (circled in box). **E** Heat map of differentially expressed genes obtained from RNA-seq of pre-invasive and invasive LUAD tumor tissues. **F** Intersection of genes identified by Venn diagram ATAC-seq and RNA-seq. **G** KEGG analysis of differentially expressed genes obtained from RNA-seq of pre-invasive LUAD compared with invasive LUAD (circled in the box). **H** MYBL2 chromatin open regions peaks are significantly more abundant in LUAD tumor tissues than in adjacent normal tissues. Peaks represent regions of enhanced gene openness, corresponding to gene promoters and transcription factor binding sites. **I** Representative IHC images of LUAD and normal tissue. **J** IHC statistical analysis showed the expression ratio of MYBL2 in LUAD and normal tissue. **K** High MYBL2 gene expression levels are associated with higher tumor stages (Figure Below). **L** PFS Kaplan–Meier survival curve based on MYBL2 expression from patients after surgery. **M** Expression levels of MYBL2 mRNA in various types of tumor tissues and normal tissues in the TIMER database. *p < 0.05, **p < 0.001, ***p < 0.0001.

Here, to identify potential master regulator transcription factors (MR-TF) driving phenotypic plasticity in LUAD, we first developed a comprehensive analysis including RNA-seq and ATAC-seq upon 5 normal tissues and 9 tumor samples (tumor samples included 4 pre-invasive LUAD and 5 invasive LUAD). ATAC-seq signals in each sample were concentrated in the transcription start site (TSS) and promoter region −2kb to 2 kb and sample library preparation was successful (Fig. 1A). RNA-seq heatmaps revealed gene differences between normal and tumor tissues (Fig. 1B). The differentially expressed genes obtained from ATAC-seq and RNA-seq were further intersected to identify common differentially regulated genes, visualized using a Venn diagram (Fig. 1C). Bioinformatics analysis (comparing normal tissue with tumor tissue) revealed that key genes overexpressed in LUAD included MYBL2, LRP1B, STK31, and several long non-coding RNAs (lncRNAs) (Fig. 1C). KEGG analysis of peak differential genes in ATAC-seq showed the differential genes were mainly involved in cell proliferation and cell death progress (circled in box) (Fig. 1D). Meanwhile, the intersection of genes, as revealed by Venn diagrams of ATAC-seq and RNA-seq, showed MYBL2 is also markedly upregulated in invasive LUAD tumors compared to pre-invasive tumors (Fig. 1E, F). KEGG analysis of differentially expressed genes between pre-invasive and invasive LUAD obtained through ATAC-seq predicted these genes participated in the biological function of cell proliferation and death (Fig. 1G). Chromatin-open regions of the MYBL2 gene were notably increased in LUAD compared to normal tissues (Fig. 1H). The peak represented the region of enhanced openness of the gene, which corresponds to the promoter and transcription factor binding site of the gene (Fig. 1H). The above data predicted MYBL2 is a potential transcription factor (TF) in LUAD tumorigenesis.

Next, immunohistochemical (IHC) staining was performed to examine the MYBL2 expression in a tissue microarray containing 67 LUAD samples and 55 normal samples. As expected, the IHC staining results clearly validated MYBL2 protein levels were higher in LUAD than normal tissues from post-operative patients of Shanghai Chest Hospital (Fig. 1I). The IHC results implied a statistically significant difference of MYBL2 levels between LUAD and normal tissues (Fig. 1J). In addition, higher MYBL2 gene RNA expression levels were associated with higher tumor stage from analysis of TCGA database (Fig. 1K). As shown in Fig. 1L, LUAD patients with high MYBL2 expression had shorter progression-free survival, with a median survival of 27.5 months in the low-expression group versus 19 months in the high-expression group. Using the TIMER platform tool, we obtained mRNA expression levels of MYBL2 across various tumor types in the TCGA and GTEx databases. MYBL2 mRNA expression is generally elevated in various types of tumors in TIMER database, and the box diagram clearly verified a remarkable increase of MYBL2 expression in LUAD compared to normal tissues (Fig. 1M). Thus, we found MYBL2 is highly expressed in LUAD, which suggests that it has the potential to be used as a biomarker to help with the diagnosis of this malignant tumour.

### MYBL2 promotes LUAD cell growth and proliferation

**Fig. 2:**
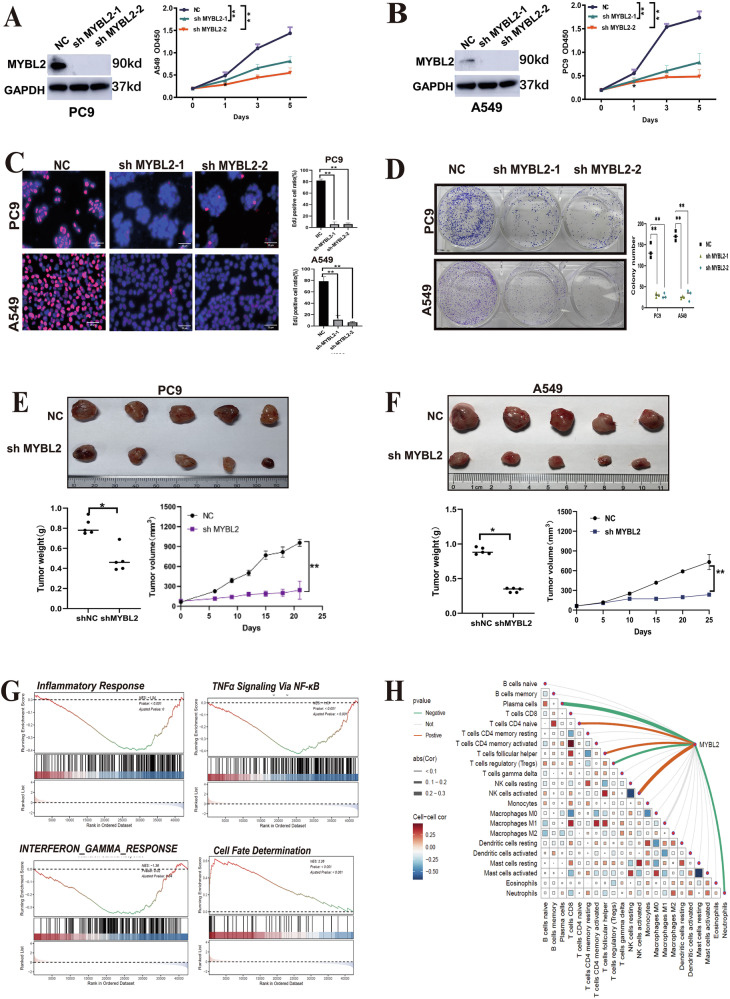
MYBL2 promotes LUAD proliferation and progression. **A**, **B** Stable shMYBL2-knockdown LUAD cell lines validated by Western blot. CCK8 assay of LUAD cells after shMYBL2 treatment. **C** TUNEL assay was used to investigate the impact of MYBL2 deficiency on LUAD. **D** Colony formation assay of LUAD cells. **E** Subcutaneous tumor formation of PC9 cells in nude mice (*n* = 5). **F** Subcutaneous tumor formation of A549 cells in nude mice (*n* = 5). **G** GSEA analysis of LUAD from the TCGA database showed MYBL2 was negatively correlated with immune inflammation pathways, including inflammatory response, NF-κB pathway, and IFN-γ factor; while the MYBL2 gene was positively correlated with cell fate determination. **H** Correlation analysis between MYBL2 and immune infiltrating cells in LUAD from the TCGA database. Data are expressed as means ± standard errors of the means of three independent experiments, *P < 0.05, **P < 0.01.

To systematically explore the biological function of MYBL2 in LUAD, lentiviral infection with shRNA plasmid was utilized to suppress MYBL2 expression in two LUAD cell lines, respectively. Immunoblotting assay verified the efficiency of shRNA (Fig. 2A, B). As expected, CCK8 assay indicated that MYBL2 knockdown (KD) significantly reduced LUAD proliferation activity (Fig. 2A, B). TUNEL assay detected a significant decrease in tumor cell death when MYBL2 was knocked down (Figs. 1C and 2C). Consistently, colony assay detected the colony formation capacity of LUAD cells was inhibited after MYBL2 KD (Fig. 2D). In order to explore the effects of MYBL2 deficiency in a living organism, the present study set up xenografts. In line with the previous in vitro findings, both volume and weight of the tumors in nude mice were significantly reduced in the shMYBL2 group compared to the control group (Fig. 2E).

Gene Set Enrichment Analysis (GSEA) from TGGA base results unveiled MYBL2 was positively involved in cell fate determination (Fig. 2F). In addition, we performed GO (Gene Ontology) analysis (Fig. 2F) and Gene-immune cell correlation analysis (Fig. 2G). The results suggested MYBL2 was negatively correlated with inflammatory response such as TNFα signaling and interferon γ response in LUAD (Fig. 2F). As is well known, pyroptosis is a distinct form of inflammatory cell death. Considering that bioinformatics analysis suggests a negative correlation between MYBL2 and inflammatory response, our research group proposed the scientific hypothesis that MYBL2 may be potentially associated with pyroptosis.

### MYBL2 inhibits GSDME-mediated cell pyroptosis in LUAD

**Fig. 3:**
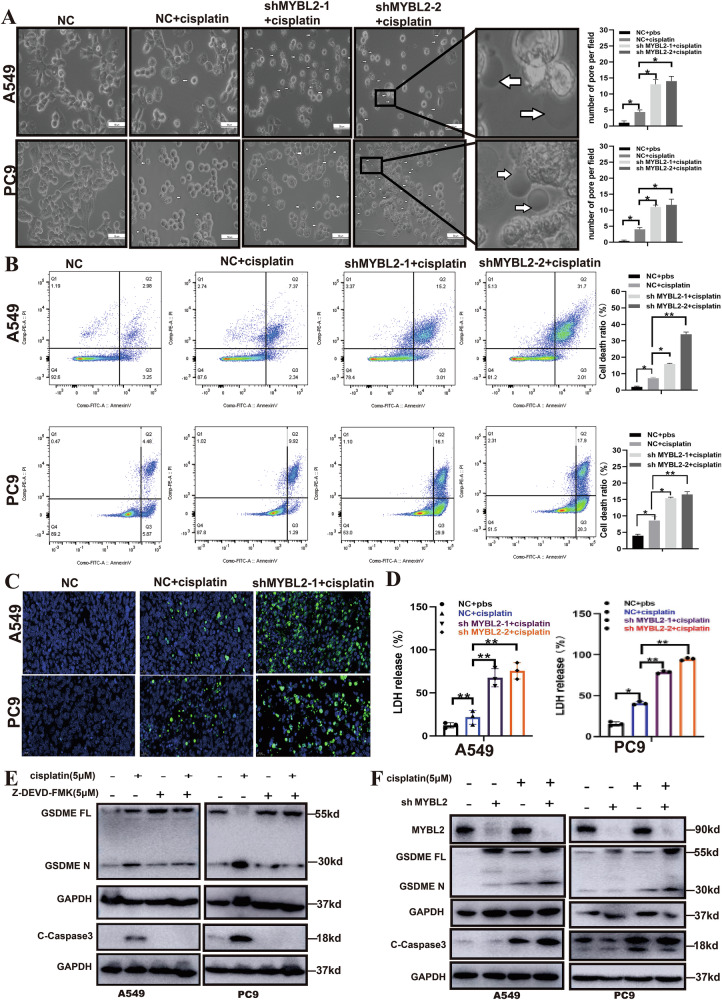
MYBL2 reduces GSDME-mediated pyroptosis in LUAD cells. **A** Representative images of LUAD cells by microscopy. (The white arrow manifests the pores originating from the plasma membrane. The scale is 50 μm). **B** Flow cytometry was used to detect cell pyroptosis. **C** TUNEL fluorescence assay was conducted detect cell pyroptosis. **D** LDH release assay was used to detect cell pyroptosis. **E** Western blot was used to examine GSDME and Cleaved-Caspase 3 protein expression after LUAD cells were treated with cisplatin (with or without Z-DEVD-FMK pretreatment). C-Caspase 3: Cleaved-Caspase 3. **F** GSDME and Cleaved-Caspase 3 protein levels were tested. Data are expressed as the mean ± standard error of three independent experiments, *P < 0.05, **P < 0.01.

Previous literatures have reported that chemotherapy drugs induced GSDME-dependent pyroptosis in various cancer types [29–31], characterized by cell swelling and cell membrane rupture. Consistently, representative bubbles of pyroptosis in A549 and PC9 cells treated with cisplatin were analyzed by transmission electron microscopy (Supplementary Fig. 1A). In this study, we found down-regulation of MYBL2 expression levels significantly promoted cisplatin-induced pyroptosis in different lung adenocarcinoma cell lines. Observation of LUAD cells after MYBL2 knockdown under a microscope indicated signs of pyroptosis. Morphologically, MYBL2 KD tumor cells displayed typical pyroptosis bubbles initiating from cell membrane. Quantitative analysis implied an increased proportion of pyroptosis in the MYBL2 KD group than the control group (Fig. 3A). In an effort to explore the underlying mechanism through which MYBL2 exerts its influence on the sensitivity of LUAD patients to the effects of cisplatin, a combination of TUNEL and Annexin V-PI assays was performed. Annexin V/PI assay showed the dead tumor cells were mainly concentrated in the Q2 quadrant (Annv^+^ PI^+^), and MYBL2 knockdown promoted cisplatin-induced tumor cell pyroptosis (Fig. 3B). TUNEL fluorescence assay results reflected that MYBL2 KD increased the percentage of tumor cell death (Fig. 3C). LDH release was also detected in LUAD cells, which is one of the features of pyroptosis. As shown in Fig. 3D, knockdown of MYBL2 increased the level of LDH release. The ELISA results showed a significant increase in cytokine levels in the cell supernatant following cisplatin treatment, compared to the untreated group. Notably, MYBL2 KD in conjunction with cisplatin treatment promoted the heightened IL-1β and IL-18 levels in the cell supernatant induced by cisplatin (Supplementary Fig. S1B–D). In addition, we found that overexpression of MYBL2 significantly inhibited cisplatin induced cell pyroptosis in lung adenocarcinoma cell lines (Supplementary Fig. 2A). Quantitative analysis showed that compared with the cisplatin only group, the MYBL2 OE group had a reduced proportion of pyroptosis in tumor cells treated with cisplatin (Supplementary Fig. 2B). As shown in Supplementary Fig. 2C, overexpression of MYBL2 decreased the level of LDH release. The ELISA results showed that MYBL2 OE inhibits an increase in IL-1β, IL-18, and HMGB1 levels in cell culture supernatants induced by cisplatin in lung cancer cell lines (Supplementary Fig. 2D). The CCK8 experiment showed that overexpression of MYBL2 reduced the sensitivity of lung adenocarcinoma cells to cisplatin chemotherapy (Supplementary Fig. 2E). The above experiments collectively suggested MYBL2 may promote cell proliferation by decreasing cellular pyroptosis.

Subsequently, western blot assay verifed that cisplatin caused caspase-3 activation in lung cancer cells, which resulted in GSDME cleavage and eventually GSDME-N fragments (Fig. 3E). Furthermore, immunoblotting experiments revealed no significant changes in the expression levels of caspase-1 and NLRP3 proteins after cisplatin stimulation, indicating that the classical pyroptosis pathway (such as the caspase-1 and NLRP3 inflammasome) was not affected (Supplementary Fig. 1E). Z-DEVD-FMK has been known as a specific and irreversible caspase-3 inhibitor. Pretreatment of tumor cells with caspase-3 inhibitor for 12 h partially blocked cisplatin-induced pyroptosis, characterized by a decrease in cleaved GSDME-N fragments. Notably, our findings suggest that inhibition of MYBL2 leads to elevation of GSDME total protein expression even without the addition of cisplatin (Fig. 3F). However, MYBL2 knockdown did not affect the caspase-3 cleavage, implying that the molecular mechanism by which MYBL2 promotes GSDME expression is independent of caspase-3 (Fig. 3F). At the same time, knockdown of MYBL2 along with cisplatin treatment of tumor cells triggered enhanced GSDME cleavage and elevated GSDME-N levels. This phenomenon led us to subsequently further excavate the molecular mechanism by which MYBL2 regulates GSDME expression.

### MYBL2 negatively regulates the expression of GSDME in LUAD

**Fig. 4:**
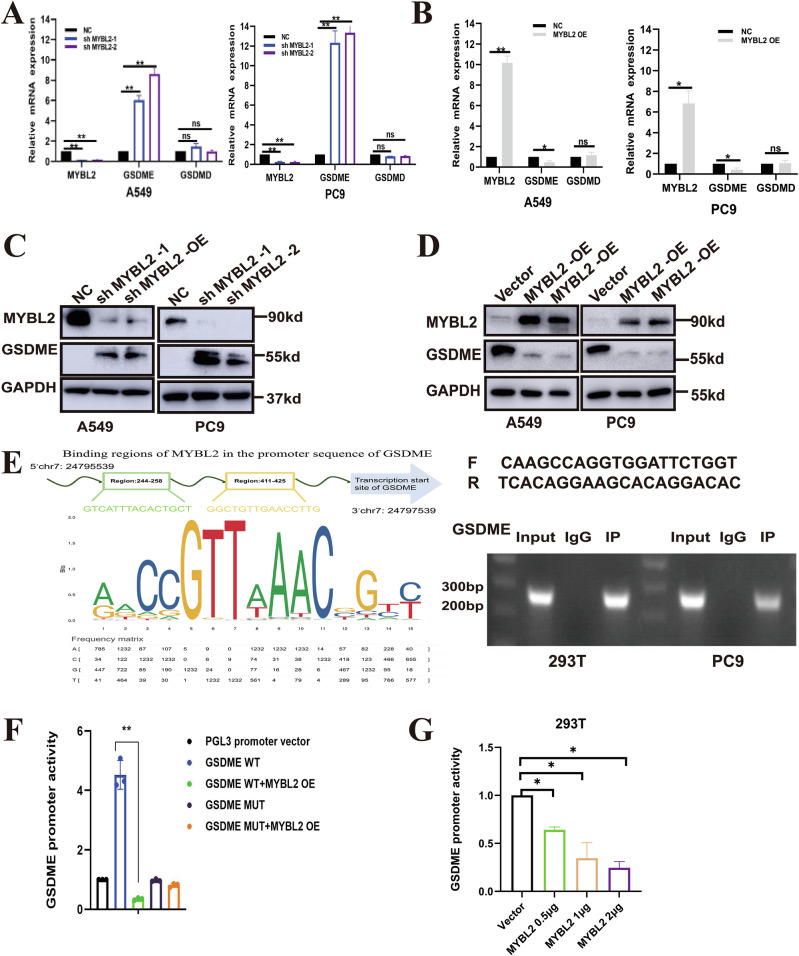
MYBL2 negatively transactivates GSDME by binding to its promoter. **A** qRT-PCR was employed to examine the effect of MYBL2 KD on GSDME RNA expression. **B** qRT-PCR was employed to examine the effect of MYBL2 OE on GSDME RNA expression. **C** Western-blot was used to verify the effect of MYBL2 KD on GSDME protein expression. **D** Western-blot was used to verify the impact of MYBL2 OE on GSDME protein expression. **E** Left panel: The binding sequences of MYBL2 to GSDME promotor obtained from the JASPAR database; Right panel: Represent image of ChIP-PCR result using MYBL2 and normal IgG pull down of chromatin using primers that amplify GSDME promoter. **F** In 293 T cells, luciferase assays were performed using co-transfection of GSDME-WT (or GSDME-Mut) promoter plasmids and MYBL2 OE plasmids (or empty vectors). **G** Luciferase assays were performed in 293 T cells. Data are expressed as the mean ± standard error of three independent experiments, *P < 0.05, **P < 0.01.

To elucidate the intrinsic mechanism of how MYBL2 repress pyroptosis in LUAD cells, the key target genes of the pyroptosis pathway were examined (GSDME, GSDMD) by qPCR experiments. The results confirmed that MYBL2 inhibition led to a significant mRNA expression increase of GSDME, whereas the knockdown of MYBL2 did not result in a change in the level of GSDMD mRNA. (Fig. 4A). Consistently, MYBL2 overexpression caused the reduction of GSDME expression levels (Fig. 4B). Consistent with the previous PCR results, the following western blot experiments verified that the knockdown of MYBL2 increased GSDME protein expression level significantly. Conversely, MYBL2 overexpression reduced the protein level of GSDME remarkedly (Fig. 4C, D).

Further bioinformatics analysis of the human GSDME promoter region has revealed the presence of a potential binding site upstream of the transcription start site (Fig. 4E left). To further investigate whether MYBL2 is recruited to the GSDME promoter, researchers performed ChIP-PCR method in 293T and PC9 cells. Primers were designed based on the predicted binding sites, and ChIP-PCR analysis verifed MYBL2 modulates GSDME by binding to the GSDME promoter region (Fig. 4E right). To confirm that MYBL2 is a key TF for GSDME transcription, we overexpressed MYBL2 gene in 293T. The level of luciferase expression originated from the GSDME promoter containing the wild-type MYBL2 binding site was significantly decreased upon MYBL2 overexpression. When this GSDME promoter mutant was transduced into 293T cells, the repression effect of MYBL2 on GSDME transcriptional activity was greatly diminished (Fig. 4F). In addition, MYBL2 negatively regulates GSDME in a dose-dependent manner, and the luciferase activity of GSDME promoter was lower when more MYBL2 plasmids were transfected (Fig. 4G). The above data implied that MYBL2 acted as the negative transcriptional regulator of GSDME.

### MYBL2 interacts with YAP1 to co-regulate GSDME expression

Previous researchers reported MYBL2 participates in the assembly of MMB complex as a core member [32, 33]. Meanwhile, genomics research by Gründl M suggests that YAP1, a core molecule of the Hippo signaling pathway, co-regulates a set of overlapping target genes in cardiomyocytes with the MMB complex [34]. However, it remains unclear whether MYBL2 is associated with YAP1 in LUAD. Based on literature review [34, 35], we proposed a hypothesis there is an interaction between MYBL2 and YAP1 that jointly regulates the transcription of GSDME, thereby inhibiting pyroptosis in LUAD and mediating cisplatin resistance.

**Fig. 5:**
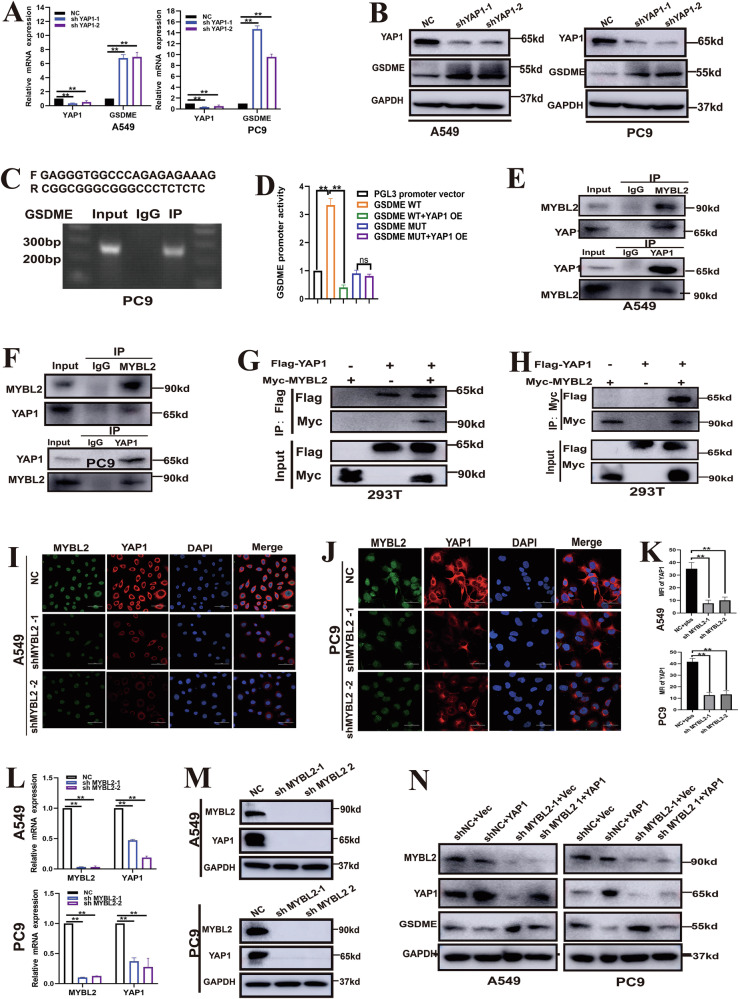
MYBL2 interacts with YAP1 to co-regulate GSDME. **A** qRT-PCR was employed to examine GSDME RNA expression. **B** Western-blot was used to verify the levels of YAP1 and GSDME protein. **C** Represent image of ChIP-PCR result using YAP1 and normal IgG pull down of chromatin using primers that amplify GSDME promoter. **D** In 293T cells, luciferase assays were performed using co-transfection of GSDME-WT (or GSDME-Mut) promoter plasmids and YAP1 plasmids (or empty vectors). **E** Interaction between MYBL2 and YAP1 was detected by endogenous Co-IP assays in A549. **F** Endogenous Co-IP assays in PC9. **G** Exogenous Co-IP assays. After co-transfection of Myc-MYBL2 and FLAG-YAP1 plasmids into 293T cells, Myc-MYBL2 protein was detected after IP of FLAG-YAP1. **H** Exogenous Co-IP assays detected interaction of Flag-YAP1 and Myc-MYBL2. **I** Immunofluorescence was conducted to analyze the MYBL2 and YAP1 levels in A549 cells. **J** Immunofluorescence was conducted in PC9 cells. **K** Quantitative analysis of the fluorescence intensity. **L** The effect of MYBL2 KD on YAP1 expression using qRT-PCR in two cell lines. **M** Detection of MYBL2 KD on YAP1 expression using immunoblotting methods. **N** Addition of YAP1 plasmids partially rescued the expression of GSDME repressed by MYBL2 deficiency in two cell lines. Data are expressed as the mean ± standard error of three independent experiments, *P < 0.05, **P < 0.01.

We first investigated the separate regulation of GSDME by YAP1. PCR method confirmed YAP1 KD obviously increased the GSDME mRNA levels (Fig. 5A). Consistent with previous PCR results, western blot experiments verified that knockdown of YAP1 significantly increased GSDME protein expression (Fig. 5B). Chip-PCR was employed to demonstrate YAP1 regulated GSDME by binding to the GSDME promoter region (Fig. 5C). Luciferase assays confirmed that luciferase levels driven by the GSDME promoter containing the wild-type YAP1 binding site were significantly reduced when YAP1 was overexpressed. When this GSDME promoter mutant was transfected into 293T cells, the inhibitory effect of YAP1 on GSDME transcriptional activity was greatly weakened (Fig. 5D). Co-IP assays showed an endogenous interaction between MYBL2 and YAP1 in two cell lines (Fig. 5E, F). This interaction was further confirmed by co-transfection of Myc-MYBL2 and Flag-YAP1 plasmids in an exogenous IP experiment (Fig. 5G, H). Furthermore, fluorescence immunocytochemistry results showed that silencing the MYBL2 gene reduced the expression level of YAP1 in LUAD cells (Fig. 5I–K). Similarly, the silencing of MYBL2 in LUAD cells was found to result in a significant reduction of YAP1 mRNA and protein levels (Fig. 5L, M). Moreover, rescue assay using western blot showed that addition of YAP1 plasmids partially rescued the expression of GSDME repressed by MYBL2 deficiency in two cell lines (Fig. 5N).

In summary, YAP1 has been confirmed to be a negative transcriptional regulator of GSDME, and MYBL2 interacts with YAP1 to co-regulate GSDME transcription.

### Upregulation of GSDME enhances pyroptosis and improves sensitivity to cisplatin

**Fig. 6:**
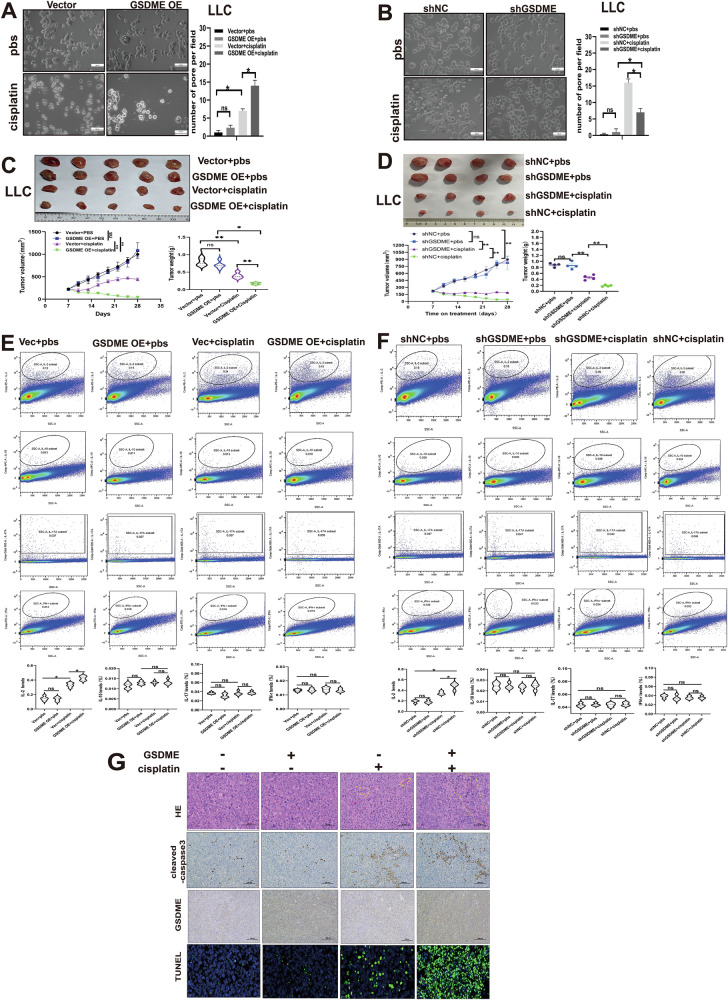
Upregulation of GSDME enhances pyroptosis and improves sensitivity to cisplatin. **A** LLC cells (LLC-Vector, LLC-GSDME) were subjected to phase contrast cell imaging. (The white arrow indicates the pores on the plasma membrane). **B** LLC cells (LLC-shNC, LLC-shGSDME) were subjected to phase contrast cell imaging. **C** Cells (LLC-Vector, LLC-GSDME) were subcutaneously injected into the C57BL/6N mice (*n* = 5). Every three days, the size of the tumor was checked. **D** Subcutaneous xenografts in C57BL/6N mice using LLC cells. Cells (LLC-Vector, LLC-shGSDME) were subcutaneously injected into the C57BL/6N mice (*n* = 4). **E** The proportion of IL-2+, IL-10+, IFN-γ+, and IL-17+ in mice tumors. **F** The proportion of IL-2+, IL-10+, IFN-γ+, and IL-17+ in mice tumors. **G** IHC and TUNEL analysis of mouse tumor tissues from LLC cells. Data are expressed as the mean ± standard error of three independent experiments, *P < 0.05, **P < 0.01.

To assess the impact of GSDME on chemotherapy sensitivity in LUAD, we constructed LLC cells that stably overexpressed GSDME or silenced GSDME. Upregulation of GSDME in LLC cells resulted in more pronounced pyroptotic bubbles, which originated from the cell membrane and led to a greater ratio of pyroptosis (Fig. 6A). In contrast, downregulation of GSDME in LLC led to the opposite effect (Fig. 6B). These in vitro experiments confirmed that GSDME OE promotes pyroptosis, thereby increasing sensitivity to chemotherapy drugs.

To identify whether GSDME affects the sensitivity of LUAD to cisplatin chemotherapy in vivo, we further constructed a xenograft mouse model. Tumor cells (LLC-Vector and LLC-GSDME) were injected subcutaneously into the right upper limb of C57 mice. Two weeks later, the cells formed tumors, and the mice were randomly separated into two groups (PBS or chemotherapy group). Tumor diameter was measured twice weekly. In the absence of cisplatin chemotherapy, no significant differences were detected in growth curves and tumor weight between the GSDME-upregulated group and the control group (Fig. 6C). However, after chemotherapy, tumors overexpressing GSDME showed significantly slower growth, smaller volume, and lower weight compared to the control group (Fig. 6C–F). Serum LDH was examined pre-chemotherapy and after-chemotherapy treatments (6G). H&E staining results implied that overexpression of GSDME in tumor sections enlarged the area of inflammatory necrosis in tumors after cisplatin chemotherapy(6H). Flow cytometry analysis of tumor-infiltrating lymphocytes (TILs) showed that T cells played a key role in cisplatin-induced tumor growth inhibition and remodeling of tumor microenvironment (TME). Specifically, we observed that, in the presence of cisplatin chemotherapy, the GSDME OE group released higher levels of components IL-2 in tumor-infiltrating lymphocytes after cisplatin induction than the control group, while the proportions of IL-10, IL17A, and IFNγ did not change (Fig. 6I and Supplementary Fig. 3). We also constructed a stable cell line that knocked down GSDME (Supplementary Fig. 4). Consistent with previous conclusions, without the involvement of cisplatin, there was no apparent difference in growth curves and tumor weight between shGSDME group and shNC group (Supplementary Fig. 4A, C). However, in the presence of cisplatin, the tumors in the shGSDME group grew faster and had larger tumor volumes compared to the shNC group (Supplementary Fig. 4A, C). Compared with the control group, the shGSDME group released lower levels of IL-2 components in tumor infiltrating lymphocytes after cisplatin induction, while the proportions of IL-10, IL-17A, and IFNγ did not change (Supplementary Fig. 4D)

These results collectively support our hypothesis that upregulation of GSDME enhances pyroptosis and improves sensitivity to cisplatin, reshaping the TME and ultimately enhancing the efficacy of chemotherapy.

### MYBL2 knockdown promotes cisplatin-mediated inhibition of LUAD proliferation in vivo

**Fig. 7:**
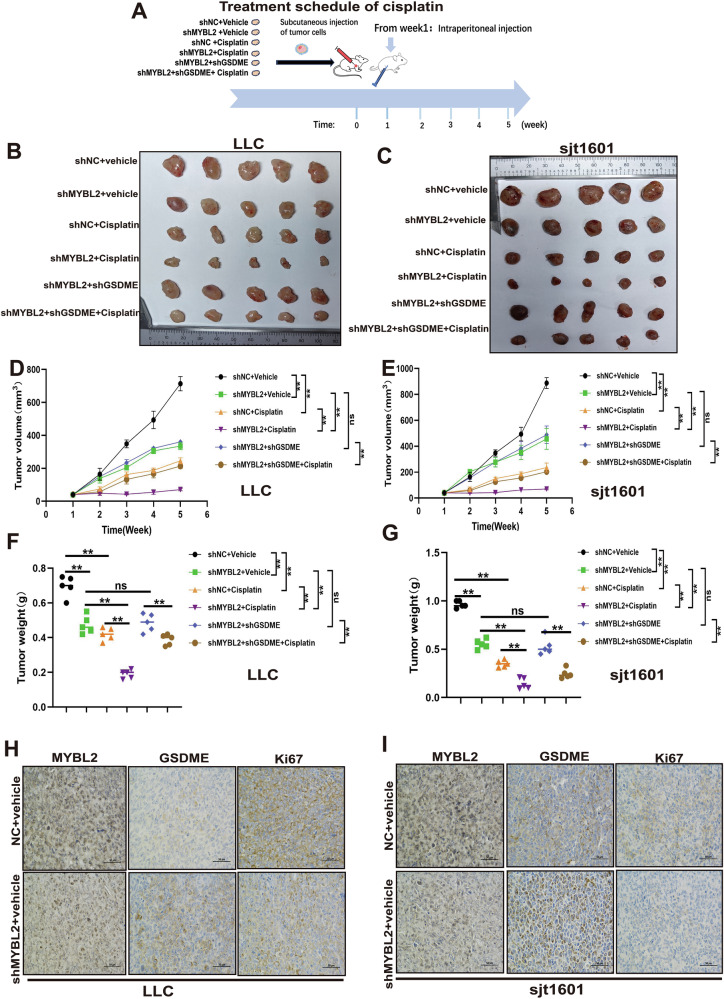
Silencing MYBL2 promotes cisplatin-mediated inhibition of LUAD proliferation. **A** Schematic view of the chemotherapy xenograft model. **B** C57BL/6N mice with LLC cells stably expressing the target gene (*n* = 5). **C** Subcutaneous xenografts in C57BL/6N mice constructed with sjt1601 cells (*n* = 5). **D** Growth curves of LLC tumors. **E** Growth curves of sjt1601 tumors. **F** Weight of the tumors in LLC cells. **G** Weight of the tumors in SJT1601 cells. **H** IHC analysis of mouse tumor tissues from LLC cells. **I** IHC analysis of mouse tumor tissues from sjt1601 cells. Data are expressed as means ± standard errors of the means of three independent experiments, *P < 0.05, **P < 0.01.

To validate the effect of the MYBL2-GSDME axis on cisplatin-induced tumor suppression, we employed a subcutaneous tumor model in C57 mice and injected mouse lung cancer cells stably expressing the target gene (LLC-shNC, LLC-shMYBL2, LLC-shMYBL2+shGSDME, sjt1601-shNC, sjt1601-shMYBL2, sjt1601-shMYBL2+shGSDME). The administration of chemotherapy drugs was conducted via the intraperitoneal route, with tumor volume measurement occurring on a biweekly basis (Fig. 7A). We observed that either knocking down MYBL2 alone or using a cisplatin-based chemotherapy regimen alone significantly inhibited lung cancer proliferation and slowed down its growth rate. In addition, the combination of MYBL2 KD and cisplatin was more effective in inhibiting tumor cell growth than cisplatin alone, and this regimen exhibited the most effective treatment results in this experiment (Fig. 7B–G). Further findings revealed that GSDME KD did not affect the inhibitory effect of MYBL2 knockdown on tumor cell proliferation in the absence of drug treatment. However, GSDME KD eliminated the growth inhibitory effect caused by the combination of MYBL2 knockdown and cisplatin treatment (Fig. 7B–G). We collected tumors and performed subsequent IHC staining. The present study demonstrated that following MYBL2 knockdown, there was an increase in the ratio of GSDME staining, accompanied by a decrease in the proportion of Ki67 staining (Fig. 7H–I), further confirming that MYBL2 knockdown can inhibit LUAD proliferation.

## Discussion

Lung cancer, a solid tumor known for high incidence and lethality, poses a major challenge to clinical treatment [36]. Cisplatin is the most commonly used chemotherapy drug in LUAD treatment, but there is still a portion of the population that cannot benefit from it, suggesting the limitations of its clinical application, so in-depth study of the resistance mechanism becomes urgent and necessary [37].

MYBL2, also known as B-Myb, belongs to the MYB transcription factor family, regulating cell cycle, cell differentiation, and cell fate [22]. Abnormal expression of MYBL2 may have tumorigenic potential and exert a significant impact on tumor by facilitating cell proliferation, treatment resistance, and metastasis. Previous studies have shown MYBL2 is overexpressed in multiple cancer types and is related to poor patient prognosis[18, 38–42]. MYBL2 has also been reported to participating in drug resistance in cancer treatment. Studies have found that in interleukin-2-dependent mouse T cells, overexpression of MYBL2 is in connection with enhanced resistance to doxorubicin, which is attributed to MYBL2’s enhanced transcriptional activation of the anti-apoptotic Bcl-2 [43]. In neuroblastoma, MYBL2 mediates tumor cell resistance to doxorubicin by regulating the expression of apolipoprotein [44]. In fibrosarcoma cells, MYBL2 is upregulated in drug resistance to DNA-interacting agents (like doxorubicin, daunorubicin, and melfalun) induced by genetic repressive elements [45]. However, MYBL2 mediates not only resistance to chemotherapy drugs but also tolerance to DNA damage, including that caused by radiation. For example, MYBL2 gene deletion leads to upregulation of the ABCG2, which in turn drives colorectal cancer resistance to chlorophyll e6-mediated photodynamic therapy [46]. Interestingly, MYBL2 was found to be chronically hypo phosphorylated, and upregulation of a non-phosphorylated MYBL2 mutant protected the neuroblastoma cells from UV-mediated death [47]. In this study, sequencing of human lung cancer tissue samples revealed that MYBL2 level was significantly higher in LUAD in comparison with normal tissue. Moreover, MYBL2 levels were remarkably higher in invasive LUAD than in pre-invasive adenocarcinoma. Our experiments confirmed MYBL2 upregulation enhances lung adenocarcinoma cell proliferation and restrains GSDME-mediated pyroptosis, thereby mediating resistance to cisplatin chemotherapy.

Recent research on the gasdermin family and pyroptosis, particularly their role in tumorigenesis and treatment resistance, has gradually become a hot research topic. PJA1 promotes drug resistance by attenuating GSDME-induced pyroptosis of nasopharyngeal carcinoma [48]. Chemotherapy drugs cause GSDME-dependent pyroptosis, and GSDME levels is positively related to PDAC initiation and chemotherapy resistance [49]. In accordance with previous reports, our research has validated that upregulating GSDME expression in lung cancer promotes pyroptosis phenotypes both in vitro and in vivo, thereby enhancing sensitivity to cisplatin drugs. It has been confirmed drugs activate Caspase 3 by damaging DNA, which subsequently mediates the cleavage of GSDME [13, 30, 50]. Western blot experiments confirmed that cisplatin can activate Caspase 3, thereby promoting GSDME cleavage and producing GSDME-N fragments in lung cancer cells. Meanwhile, this cleavage process can be blocked by a Caspase-3 inhibitor. This study found that MYBL2 KD increased GSDME protein levels without interfering with caspase 3 activation, leading to elevated GSDME levels in the absence of cisplatin treatment. Additionally, knockdown of MYBL2 along with cisplatin treatment of tumor cells triggered enhanced GSDME cleavage and elevated GSDME-N levels.

Furthermore, the DREAM complex, composed of p130/107, DP1/2, E2F4/5, and MuvB core, inhibits the transcription of cell cycle genes under physiological conditions [51]. The absence of DREAM, in conjunction with the abnormal expression of MYBL2, MuvB, and FOXM1, is frequently observed in neoplastic lesions. It is important to note that the YAP1 protein, which is associated with castration-resistant prostate cancer, has been observed to cooperate with the Myb-MuvB complex. And this cooperation has been found to induce mitosis-related gene expression, thereby promoting the progression of liver cancer [52]. However, in lung adenocarcinoma, the relationship between MYBL2 and YAP, especially with GSDME, has not been reported. Interestingly, we found that MYBL2 negatively regulates GSDME by directly binding to its promoter. Furthermore, MYBL2 interacts with YAP1 to negatively regulate GSDME transcription, thereby inhibiting pyroptosis and mediating cisplatin chemotherapy resistance in LUAD cells. Therefore, targeting the MYBL2/GSDME axis may be a promising strategy to make LUAD sensitive to chemotherapy.

Although this work has confirmed that MYBL2 negatively regulates GSDME expression in LUAD cells, the complex cross-regulatory mechanisms by which MYBL2 modulates pyroptosis remain to be explored. Further experiments are needed to investigate other biological functions of MYBL2 in LUAD. Additionally, pyroptosis, a form of cell death characterized by its pro-inflammatory properties, has been observed to activate anti-tumor immune responses and enhance the chemosensitivity of tumors. Further validation in clinical, cellular, and animal experiments is required to determine whether and how MYBL2 mediates drug resistance by inducing an immunosuppressive microenvironment. Large-scale immunological experiments and lung adenocarcinoma patient samples are required to identify specific antigens and inflammatory cytokines released during chemotherapy-induced tumor cell pyroptosis. This will provide guidance for overcoming drug resistance and identifying LUAD patients who may benefit from combined chemotherapy and immunotherapy in the future.

## Conclusion

In the present study, it was demonstrated that the transcription factor MYBL2 exerts a negative regulatory influence on the GSDME gene, thereby modulating the process of pyroptosis and attenuates the sensitivity of lung cancer cells to cisplatin treatment. The results of this research have potential clinical translational value as a scientific basis for new strategies for subsequent screening of effective populations and overcoming treatment resistance.

## Materials and methods

### Clinical specimens

Human lung cancer samples were achieved from Shanghai Chest Hospital, Shanghai Jiao Tong University School of Medicine (Shanghai, China) after surgical resection. Our research group obtained written informed consent from cancer patients for the use of their tissue samples for research purposes. The Ethics Committee of the Research Institute of Shanghai Chest Hospital approved this study (Approval No. KS25267).

### Cell culture and reagents

A549 (lung adenocarcinoma, human), PC9 (lung adenocarcinoma, human), and LLC (Lewis lung carcinoma, mouse) cell lines were bought from the ATCC (USA). SJT1601 cell on a C57BL/6 background were kindly provided by Prof. Deng from Shanghai Jiao Tong University School of Medicine [53–55]. Cells were cultured in either RPMI or DMEM (both from Corning) with 10% FBS (from Gibco, Grand Island, NY, USA), as well as penicillin and streptomycin (both from Gibco, Grand Island, NY, USA). All cells were cultured at 37 °C in a humidified incubator with 5% CO₂. To evoke cell pyroptosis, cells were treated in a serum-free medium and then processed with cisplatin (Selleck) preincubated with or without Z-DEVD-FMK (Selleck).

### ATAC-seq and RNA-seq

The construction of ATAC-seq libraries involves three steps: nucleus preparation, translocation, and amplification. First, tissues or cells to be examined are suspended as intact, homogeneous single cells and subsequently incubated in lysis buffer to produce crude nuclei. Second, the suspended nuclei are incubated in the translocation reaction mixture to produce DNA fragments. Finally, the transposed DNA is amplified to generate a sequencing library. RNA sequencing library construction mainly includes the following steps: RNA extraction, polyA strand-specific library construction, after Bioptic Qsep100 quality control, and sequencing using Illumina high-throughput sequencing platform (NovaSeq 6000).

### Immunohistochemistry (IHC)

Tissue samples were obtained from patients diagnosed as LUAD from Shanghai Chest Hospital, Shanghai Jiao Tong University School of Medicine. All participants have signed informed consent forms. IHC staining of tumor sections was conducted following the manufacturer’s directions. Briefly, tissues were deparaffinized, extracted, and stained with antibodies. The staining intensity was categorized as: 1 (0–25%), 2 (26–50%), 3 (51–75%), and 4 (76–100%). The intensity score and percentage score of IHC staining are multiplied to obtain the final score. IHC staining scores were determined independently under identical conditions by two specialists who maintained confidentiality of patients’ clinical and prognostic information. In vivo, immunohistochemistry of mouse tumors was performed as before. Primary antibodies were diluted as follows: GSDME, 1:100; cleaved-caspase3, 1:100; Ki67, 1:100; MYBL2, 1:100.

### Gene transfection and knockdown

Human MYBL2 and YAP1 plasmids were synthesized and cloned into pcDNA3.1 vector (Genepharma, Suzhou, China). The PGL3 promoter vector plasmid (GSDME-WT) and the mutant plasmid (GSDME-Mut) were purchased from Genepharma. Plasmids were transfected into A549 and PC9 cells using Lipofectamine 3000 (Invitrogen, USA). A stable lung cancer cell line was obtained by infecting lung cancer cells with a short hairpin RNA lentivirus (shMYBL2 and shYAP1) (Hanbio, Shanghai, China).

### RNA extraction and qRT-PCR

Add 1 mL of Trizol (Vazyme, Nanjing, China) to every 5–10 × 10⁶ cells and use Trizol to disrupt the cells and release the RNA. RNA was transcribed into complementary DNA using the Prime Script RT Reagent Kit (Servicebio, Beijing, China). SYBR Premix Ex Taq (Servicebio, Beijing, China) was used to define mRNA level, and ACTIN acted as a reference gene. Primer sequences are listed in Supplementary Table S1.

### Western blot (WB) and Immunoprecipitation (IP)

Adherent cells were lysed with RIPA or IP lysate containing PMSF and protease inhibitors (Beyotime Biotechnology, Shanghai, China). Cells were immunoprecipitated using Protein A/G Immunoprecipitation Kit (Roche, Switzerland). At 4 °C, the cell lysate was incubated with the primary antibody and Protein A/G beads. The beads are washed and eluted with 1× Sample at 100 °C before being examined by Western blot. To minimize the interference of IgG heavy chain, a specific heavy chain avoidance secondary antibody (Abbkine, China) was used in the WB analysis to avoid such cross-reactions. Anti-cleaved caspase-3 (Abcam, England), anti-cleaved caspase-1 (Abcam, England), anti-caspase-1 (Abcam, England), anti-NLRP3 (Abcam, England),anti-GSDME (Abcam, England), anti-YAP1 (CST, USA), anti- MYBL2 (Proteintech, China), Myc tag (Abmart, China), Flag tag (Abmart, China), anti-Tubulin (CST, USA), and anti-GAPDH antibody (CST, USA) were introduced as the first antibodies to detect the target protein. The HRP-conjugated secondary antibody, namely Goat anti-rabbit, was procured from CST, USA.

### Immunofluorescence staining and confocal fluorescence microscopy photography

Tumor cells were digested and planted in six-well plates prepped for crawlers. After a certain period of time, the culture solution in the cell culture plate was aspirated and then washed softly three times with PBS. Tumor cells on the crawls were fixed with 4% paraformaldehyde and softly washed 3 times with PBS. After blocking the slides with BSA, the primary antibody and species-specific fluorescent secondary antibody were incubated sequentially according to the procedure. Finally, the slides were stained for nuclei with DAPI and photographed under a confocal fluorescence microscope.

### Transmission electron microscope (TEM)

Firstly, we treated lung cancer cell lines with a certain concentration of cisplatin and fixed them to maintain their morphological structure. Then perform dehydration treatment and gradually replace water with organic solvents. Next, immerse the cell sample in a suitable resin for impregnation and embedding. Subsequently, the fixed cell samples were sliced into extremely thin sections and transferred onto a copper grid. Finally, use TEM to observe and capture the pores and bubbles generated during the defocusing process.

### Chromatin immunoprecipitation PCR (ChIP-PCR)

ChIP-PCR was conducted using the Pierce Agarose ChIP Kit (Thermo, USA) and Premix Taq™ (Takara, Japan) following the manufacturer’s directions. In short, crosslinking of proteins with DNA was achieved by treating the cell cultures with 1% formaldehyde. Following glycine termination of cross-linking, the cells were collected, and chromatin was broken down by sonication. A total of 4 μl of anti-MYBL2 antibody (Proteintech, Wuhan, China) or (Proteintech, Wuhan, China) was added to the fragmented chromatin and protein A/G beads in a rotating incubator at 4 °C overnight. The process of immunoprecipitation was followed by washing the resultant chromatin with low-salt and high-salt ChIP buffers. The experimenters finally performed qRT-PCR and calculated the efficiency of IP as the percentage of fragmented DNA relative to the input. The experiment was conducted at least three times.

### Lactate dehydrogenase (LDH) release assay

LDH activity measurement was performed by LDH Cytotoxicity Assay Kit (Beyotime Biotechnology, Shanghai, China), following the collection of cell culture Supernatant. LDH levels of the cell culture supernatant were normalized against the levels in the control group’s culture medium. The microplate spectrophotometer (BioRad) was used to quantify the absorption at 490 nm. Experiment was carried out at least three occasions.

### Cell viability

Cells were transfected with plasmids knocking down MYBL2 or control vectors according to the instructions, and then cells were inoculated into 100 μL of culture medium in 96-well cell culture plates at a concentration of 3000 cells per well. After the incubation period, CCK8 solution (Beyotime Biotechnology, Shanghai, China) was added following the removal of the medium. The cells were incubated, after which the absorbance was measured. All experiments were performed in three independent experiments.

### Colony formation assays

Cells were seeded at a concentration of 1000 cells per well in 6-well plates. The cells were cultured in serum-free RPMI/DMEM, and the medium was changed biweekly. After a period of time, the colonies were fixed in a 10% formalin solution following removement of the medium. They were stained with a 0.1% crystal violet solution for 10 min. Colony counting was performed using ImageJ software, and a digital image of the plate was taken.

### TUNEL assay

Cell slides were fixed with formaldehyde. Subsequently, TUNEL assay was applied in accordance with the manufacturer’s procedures. The cell slides were then subjected to visualization via the use of a fluorescence microscope.

### Dual-luciferase reporter assay

The procedure involved cloning the wild-type (WT) or mutant (Mut) full-length human GSDME promoter sequence into pLenti-Promoter-Dual-Luc vectors. All plasmids were purchased from Genepharma, Suzhou, China. A GSDME luciferase reporter was used in transient co-transfection (either WT or Mut, including a Renilla vector control) using lipo3000 (Invitrogen, USA). Following the incubation period, cells were assessed by the Dual-Lumi™ Luciferase Reporter Gene Assay Kit (Beyotime Biotechnology, Shanghai, China). Experiments were conducted at least three times.

### Flow cytometry analysis and cell marker staining

Tumor cells were digested, collected, and labeled with Annexin V-FITC /PI kit (BD Biosciences). After incubation for 5 min, each sample was added into 1× binding buffer, and data were acquired by flow cytometer (BD Biosciences). Tumor tissue samples were digested, collected and stained with 1 μL/sample fluorochrome-labeled antibodies of CD45, CD3, CD8, CD44, CD62L, Live/dead, PD-1, IFN-γ, IL-2, IL-10and IL-17 were purchased from BD, USA.

### Xenograft tumor model

Lung cancer cells were injected into the right upper extremity axilla of C57BL/6N mice or nude mice, as described with slight modifications. Cisplatin or PBS was applied every three days by intraperitoneal injection (10 mg/kg/mouse). The tumor size was measured using a caliper, and tumor volume was calculated using the formula (length × width × width)/2. Animals were kept in a specific pathogen-free (SPF) Animal Center following the Laboratory regulations approved by the Institutional Ethics Committee of Shanghai Chest Hospital, Jiao Tong University.

### Gene expression analysis in GEPIA

In our study, we utilized databases to analyze gene expression and staging correlations. GEPIA (URL: http://gepia.cancerpku.cn/index.html).

### Statistical analysis

Statistical analysis was performed using GraphPad Prism software (GraphPad Software, San Diego, CA). The Student’s *t*-test was utilized in order to compare the differences. Data is expressed as the mean ± SD of a minimum of three independent experiments. In the context of statistical analysis, a *P*-value of less than 0.05 was considered to be significant.

## Supplementary information

Supplementary Fig.1

Supplementary Fig.2

Supplementary Fig.3

Supplementary Fig.4

supplementary Table1

Supplementary Figure ligends

original data

Fig 2F original data

Fig 2G original data

Fig 1D original data

Fig 1G original data

Reproducibility Checklist

## Data Availability

The original findings presented in this research are included in this article. Further questions can be directed to the corresponding author.
