# Supplementary material for: MYBL2 impedes cisplatin sensitivity through suppressing GSDME-mediated pyroptosis in lung adenocarcinoma
Source: Cell Death Discov. 2026 Jun 2;12:327. doi: 10.1038/s41420-026-03175-y (PMC13443601; doi:10.1038/s41420-026-03175-y)
Supplement: Supplementary file 2 [file 41420_2026_3175_MOESM6_ESM.pdf]

Supplementary Figure Legends

Figure S1. Cisplatin can induce cell pyroptosis in lung adenocarcinoma cell lines.

A. Representative bubbles of pyroptosis in A549 and PC9 cells treated with cisplatin were analyzed by transmission electron microscopy.

B-D. MYBL2 KD in conjunction with cisplatin treatment promoted the heightened IL-1β and IL-18 levels in the cell supernatant induced by cisplatin.

E. Immunoblotting experiments revealed no significant changes in the expression levels of caspase-1 and NLRP3 proteins after cisplatin stimulation.

Data are expressed as means ± standard errors of the means of three independent experiments, $*P < 0.05$, $**P < 0.01$.

Figure S2. Overexpression of MYBL2 significantly inhibited cisplatin induced cell pyroptosis in lung adenocarcinoma cell lines.

A. Representative bubbles of pyroptosis in A549 and PC9 cells.

B. Quantitative analysis showed that compared with the cisplatin only group, the MYBL2 OE group had a reduced proportion of pyroptosis in tumor cells treated with cisplatin.

C. Overexpression of MYBL2 decreased the level of LDH release.

D. The ELISA results showed that MYBL2 OE inhibits an increase in IL-1β, IL-18 and HMGB1 levels in cell culture supernatants induced by cisplatin in lung cancer cell lines.

E. The CCK8 experiment showed that overexpression of MYBL2 reduced the sensitivity of lung adenocarcinoma cells to cisplatin chemotherapy.

Data are expressed as means ± standard errors of the means of three independent experiments, $*P < 0.05$, $**P < 0.01$.

Figure S3. Compared with the chemotherapy-only group, the GSDME OE group released higher levels of components IL-2 in tumor-infiltrating lymphocytes after cisplatin induction

A. Diagram of flow cytometry gating strategies for TILs.

B. The proportion of IL-2， IL-10, IL-17, IFN-γ + in tumor-infiltrating immune cells in each group（$n = 5$）.

Data are expressed as means ± standard errors of the means of three independent experiments, *P < 0.05, **P < 0.01.

Figure S4. In the presence of chemotherapy, tumors with GSDME knockdown grew faster than those in the control group.

A. Subcutaneous xenografts in C57BL/6N mice using LLC cells. Cells (LLC-Vector, LLC-shGSDME) were subcutaneously injected into the C57BL/6N mice (n = 4).

B. Growth curves of the tumors using LLC cells.

C. Weight of the tumors in LLC cells.

D. The proportion of IL-2， IL-10, IL-17, IFN-γ + in TILs.

Data are expressed as means ± standard errors of the means of three independent experiments, *P < 0.05, **P < 0.01.
