# Supplementary material for: MYBL2 impedes cisplatin sensitivity through suppressing GSDME-mediated pyroptosis in lung adenocarcinoma
Source: Cell Death Discov. 2026 Jun 2;12:327. doi: 10.1038/s41420-026-03175-y (PMC13443601; doi:10.1038/s41420-026-03175-y)
Supplement: Supplementary file 3 [file 41420_2026_3175_MOESM7_ESM.pdf]

Figure 2A-B

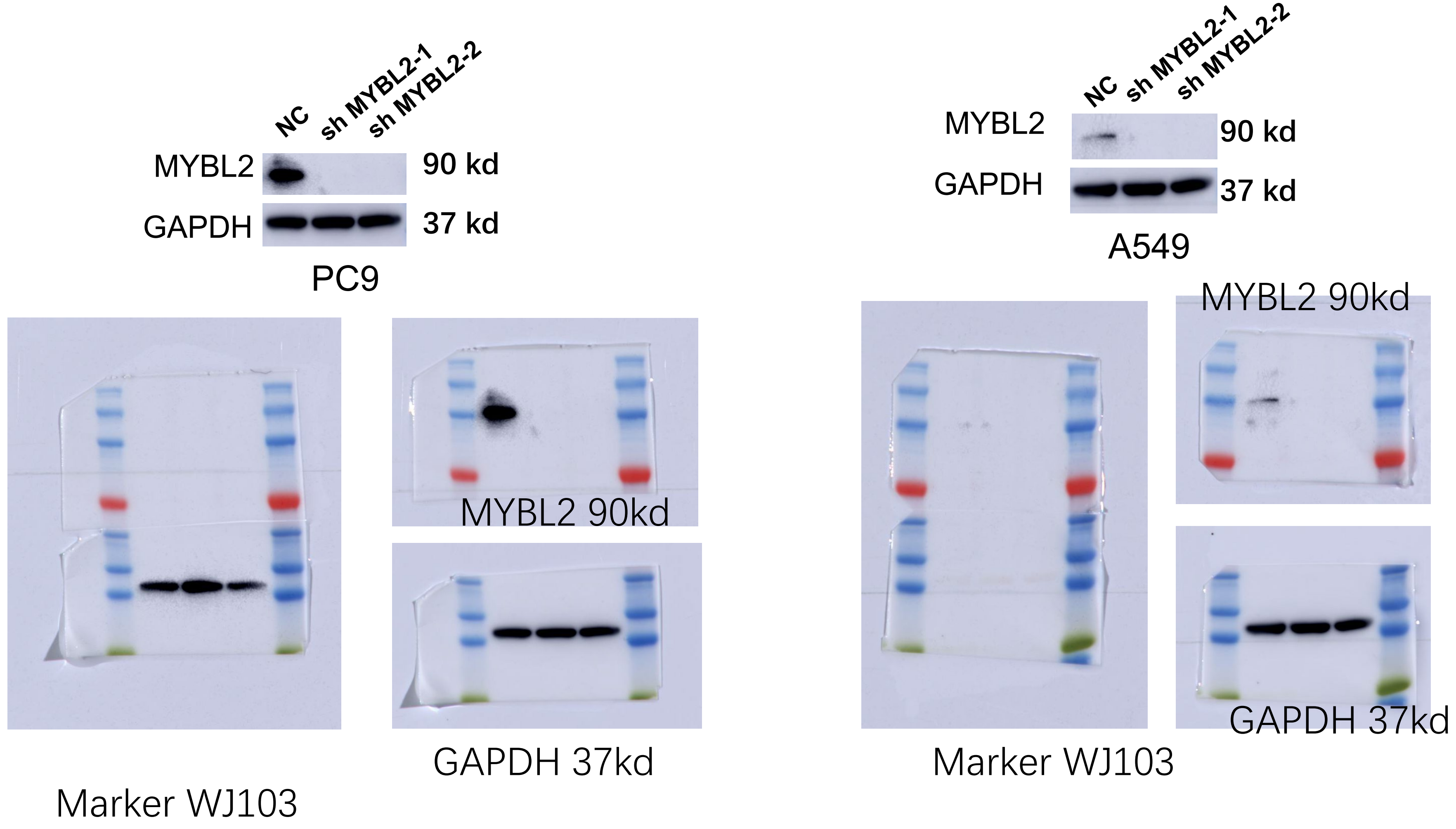

NC
sh MYBL2-1
sh MYBL2-2
MYBL2
90 kd
GAPDH
37 kd
PC9
MYBL2 90kd
GAPDH 37kd
Marker WJ103
NC
sh MYBL2-1
sh MYBL2-2
MYBL2
90 kd
GAPDH
37 kd
A549
MYBL2 90kd
GAPDH 37kd
Marker WJ103

Figure 3E

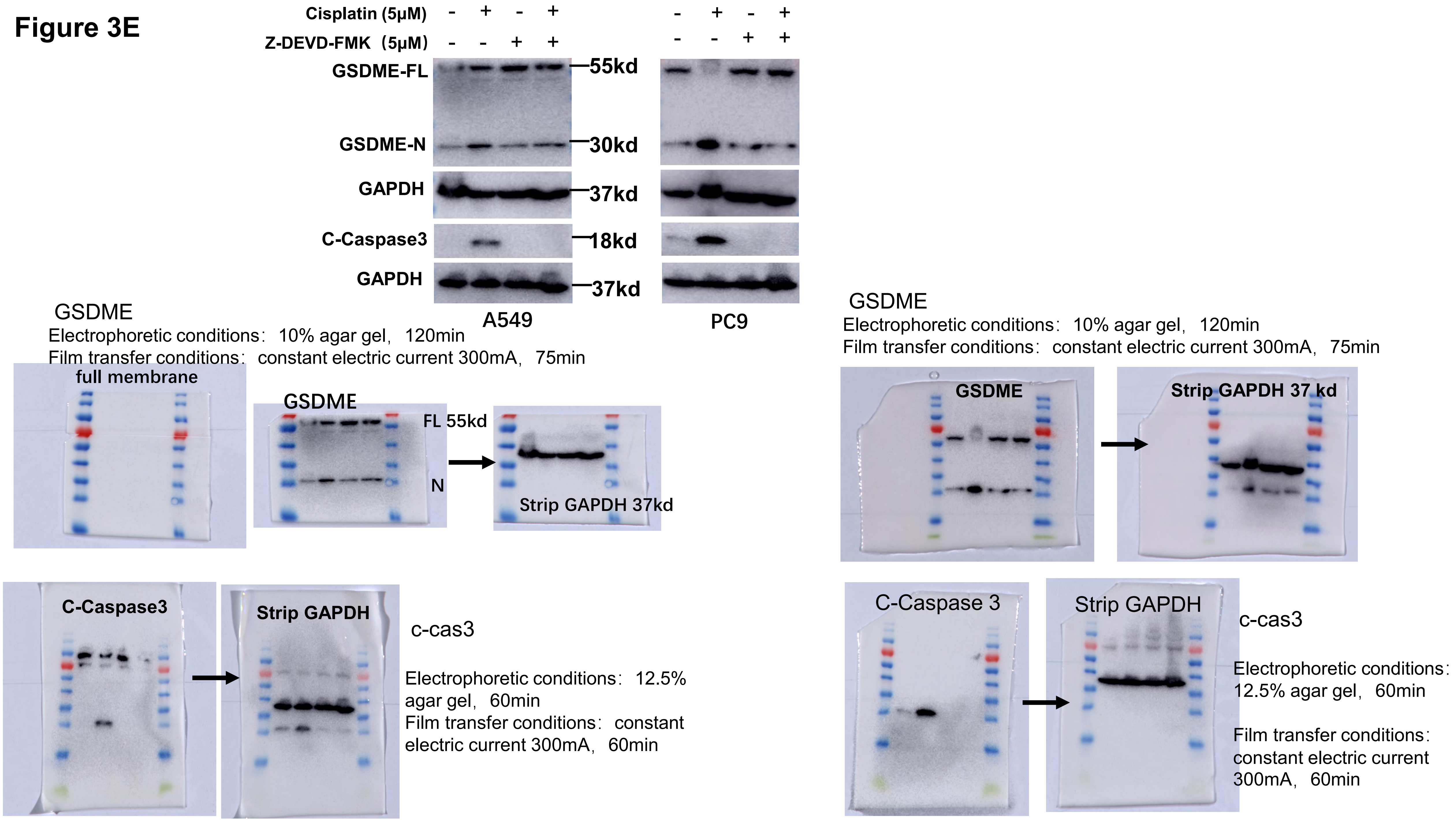

Cisplatin (5μM) - + - + - + - +
Z-DEVD-FMK (5μM) - - + + - - + +
GSDME-FL
55kd
GSDME-N
30kd
GAPDH
37kd
C-Caspase3
18kd
GAPDH
37kd
A549
PC9
GSDME
Electrophoretic conditions: 10% agar gel, 120min
Film transfer conditions: constant electric current 300mA, 75min
full membrane
GSDME
FL 55kd
N
Strip GAPDH 37kd
C-Caspase3
Strip GAPDH
c-cas3
Electrophoretic conditions: 12.5% agar gel, 60min
Film transfer conditions: constant electric current 300mA, 60min
GSDME
Electrophoretic conditions: 10% agar gel, 120min
Film transfer conditions: constant electric current 300mA, 75min
GSDME
Strip GAPDH 37 kd
C-Caspase 3
Strip GAPDH
c-cas3
Electrophoretic conditions: 12.5% agar gel, 60min
Film transfer conditions: constant electric current 300mA, 60min

Figure 3F left

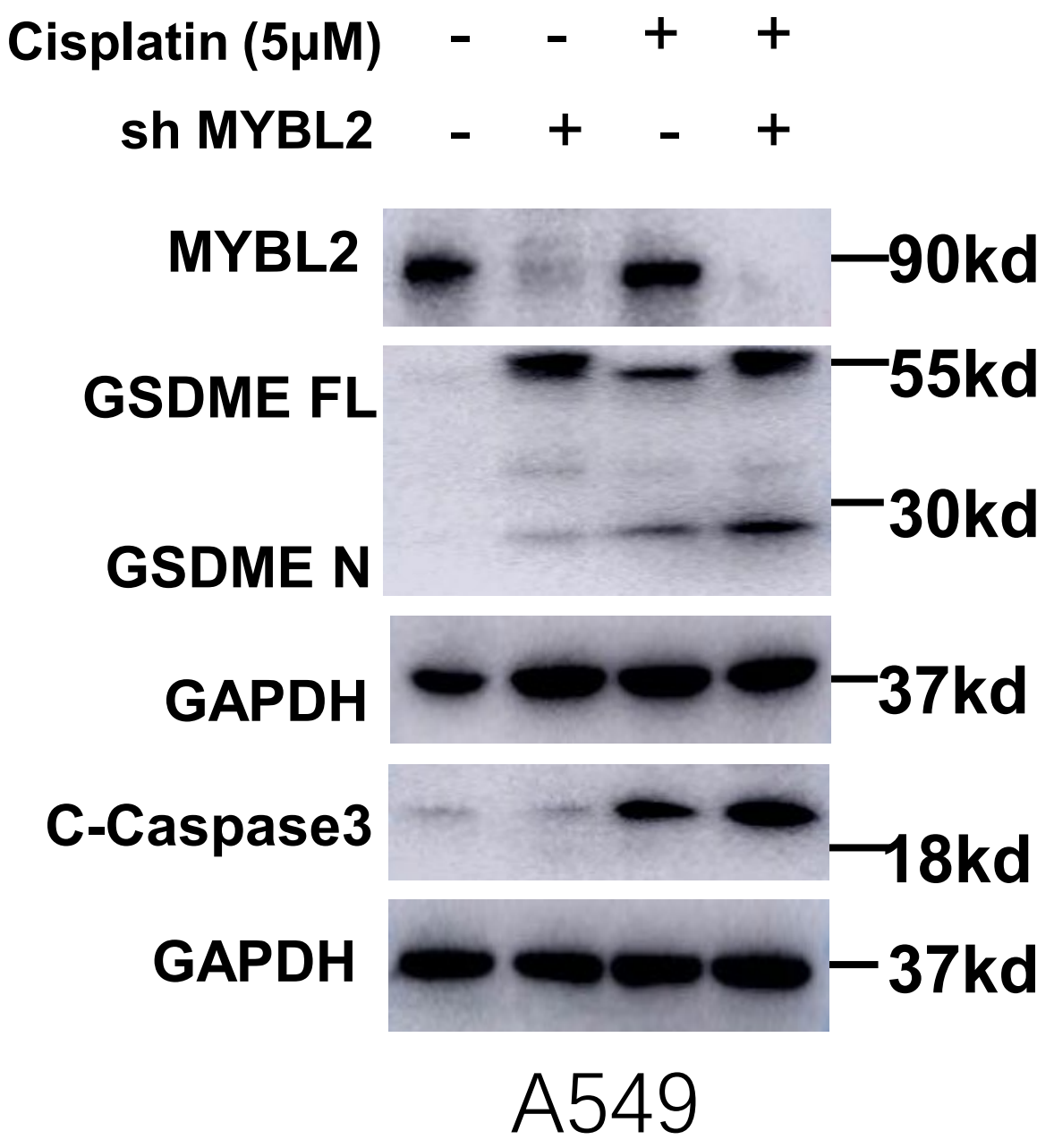

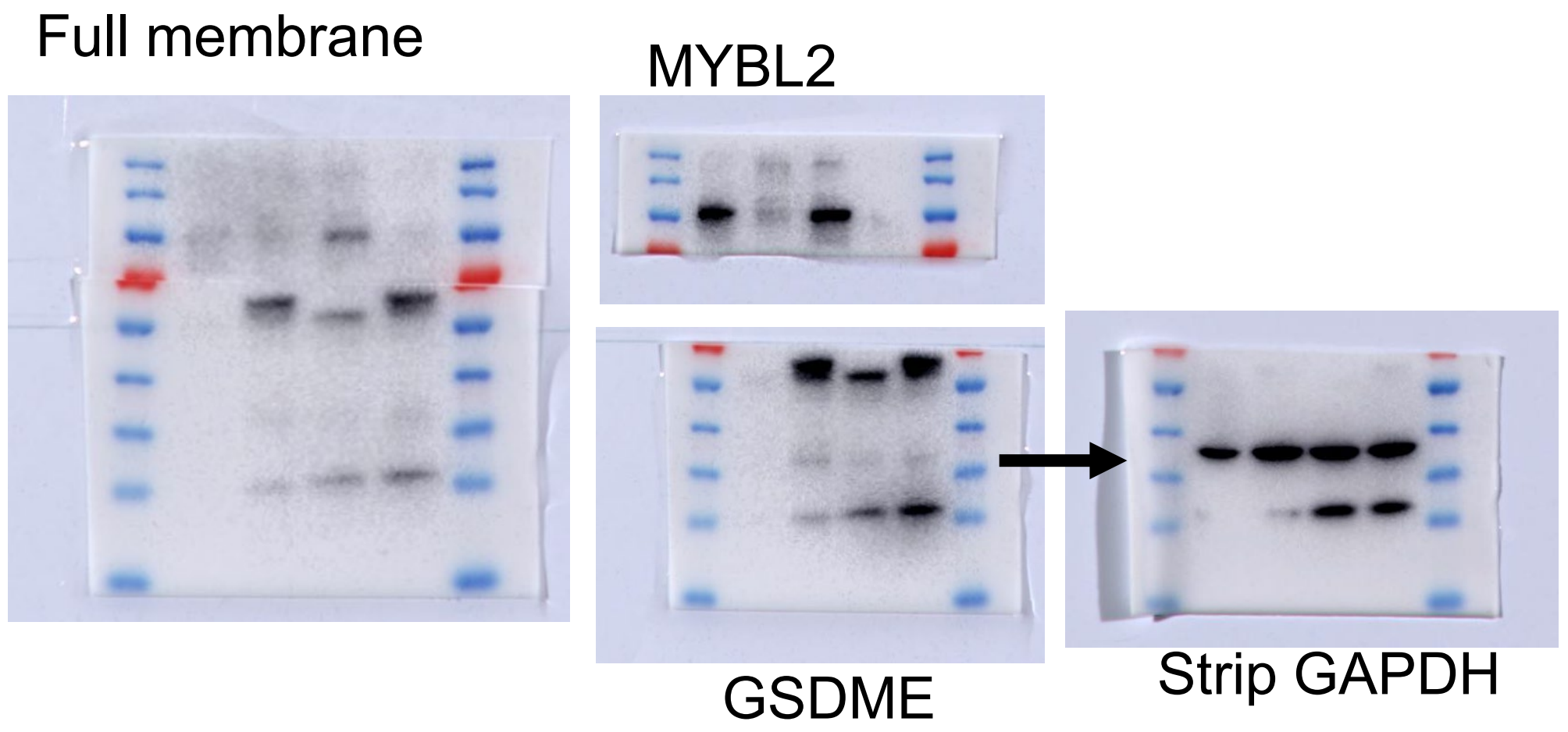

Experimental condition:
Electrophoretic conditions: 10% agar gel, 120min
Film transfer conditions: constant electric current 300mA, 75min

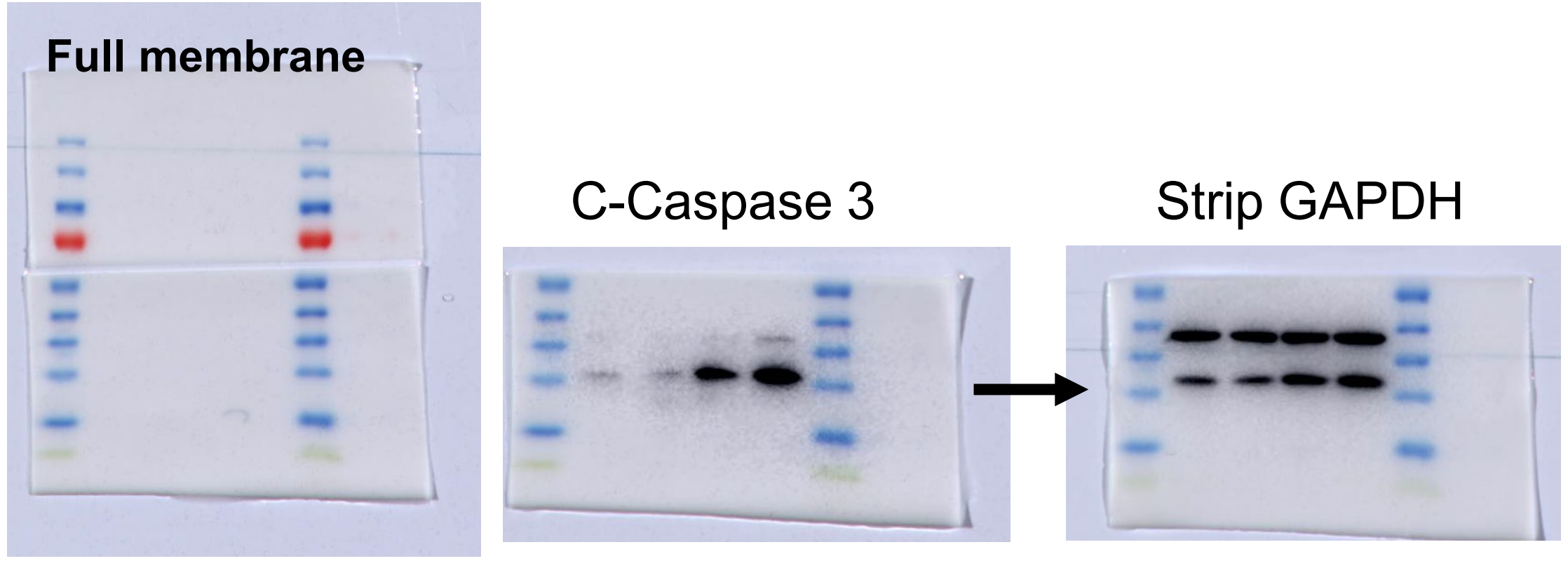

Experimental condition:
Electrophoretic conditions: 12.5% agar gel, 60min
Film transfer conditions: constant electric current 300mA, 60min

Figure 3F right

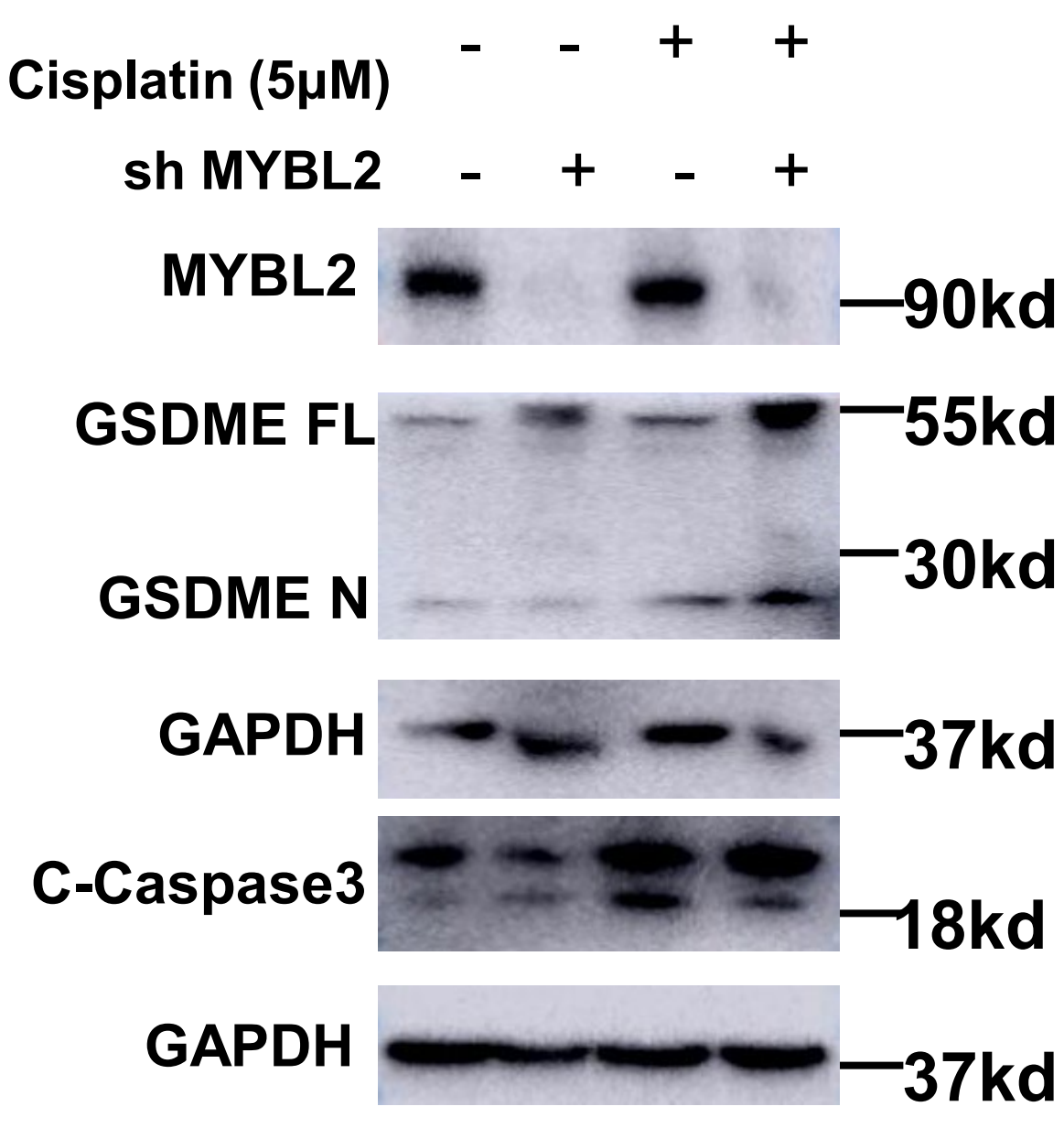

PC9

Marker 26616

Experimental condition:
Electrophoretic conditions: 10% agar gel, 120min
Film transfer conditions: constant electric current 300mA, 75min

Full membrane

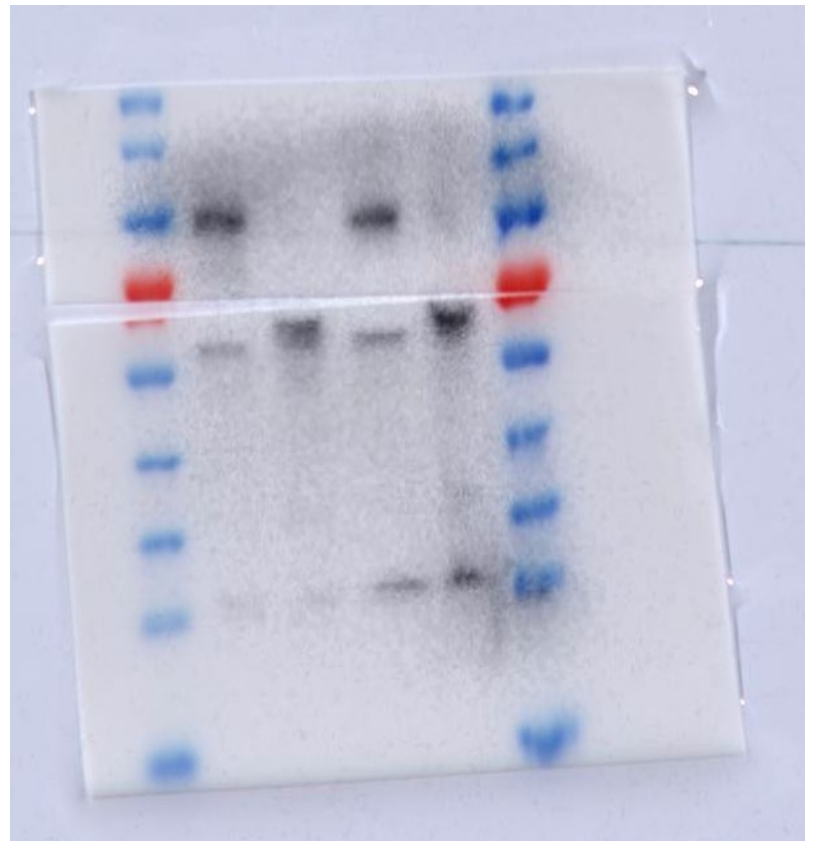

MYBL2

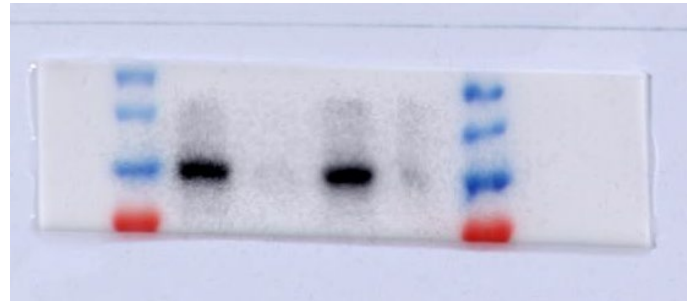

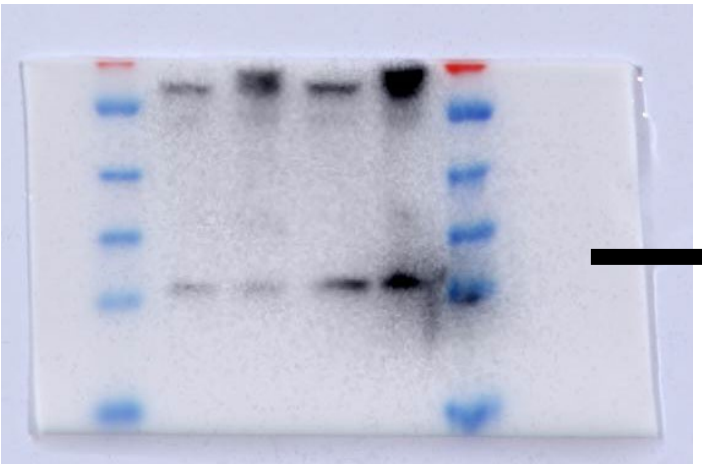

GSDME

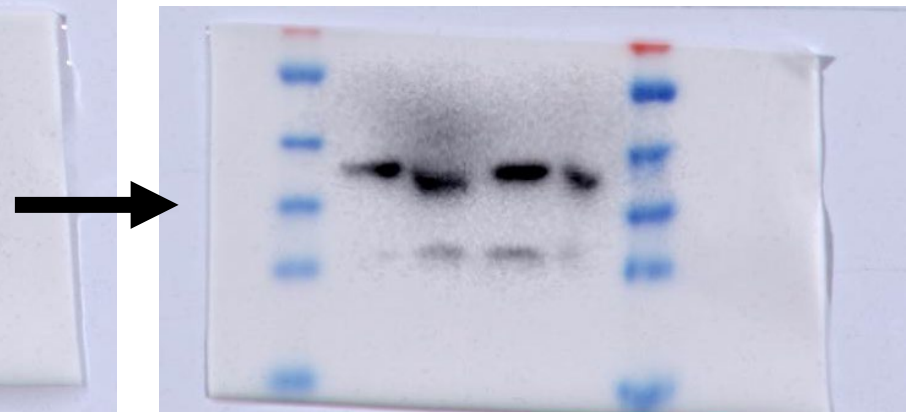

Strip GAPDH

Experimental condition:
Electrophoretic conditions: 12.5% agar gel, 60min
Film transfer conditions: constant electric current 300mA, 60min

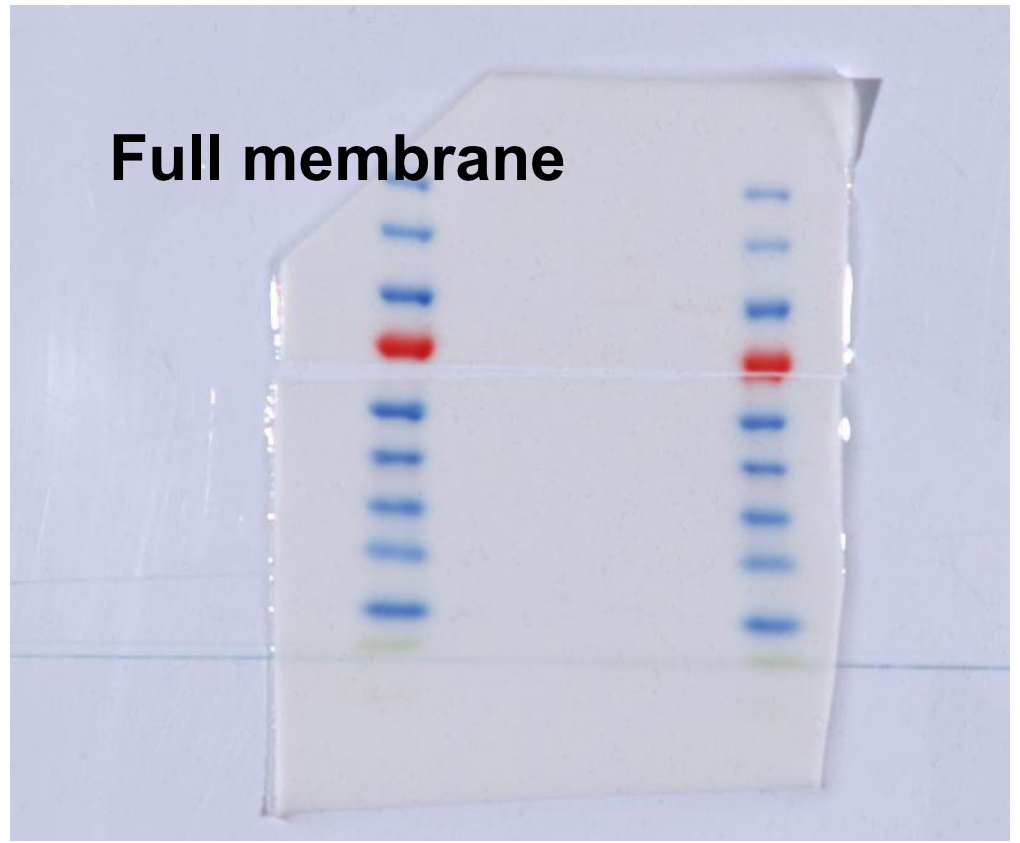

C-Caspase 3

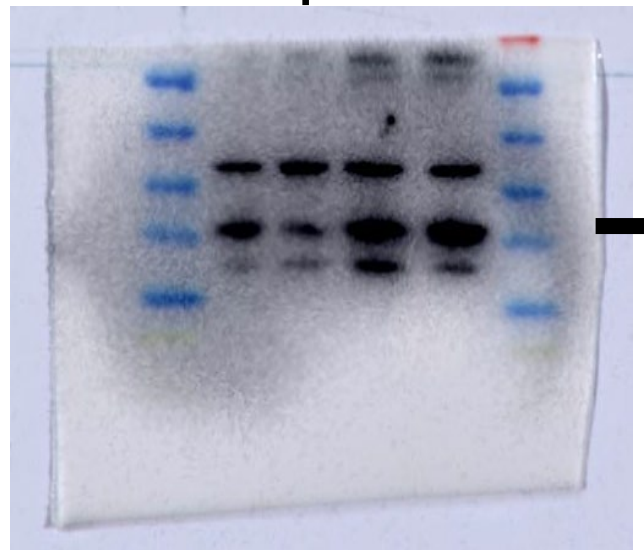

Strip GAPDH

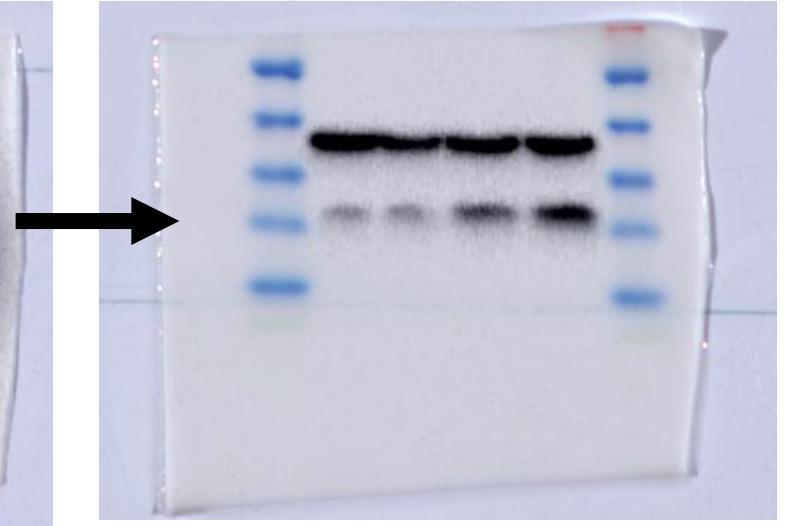

Figure 4C

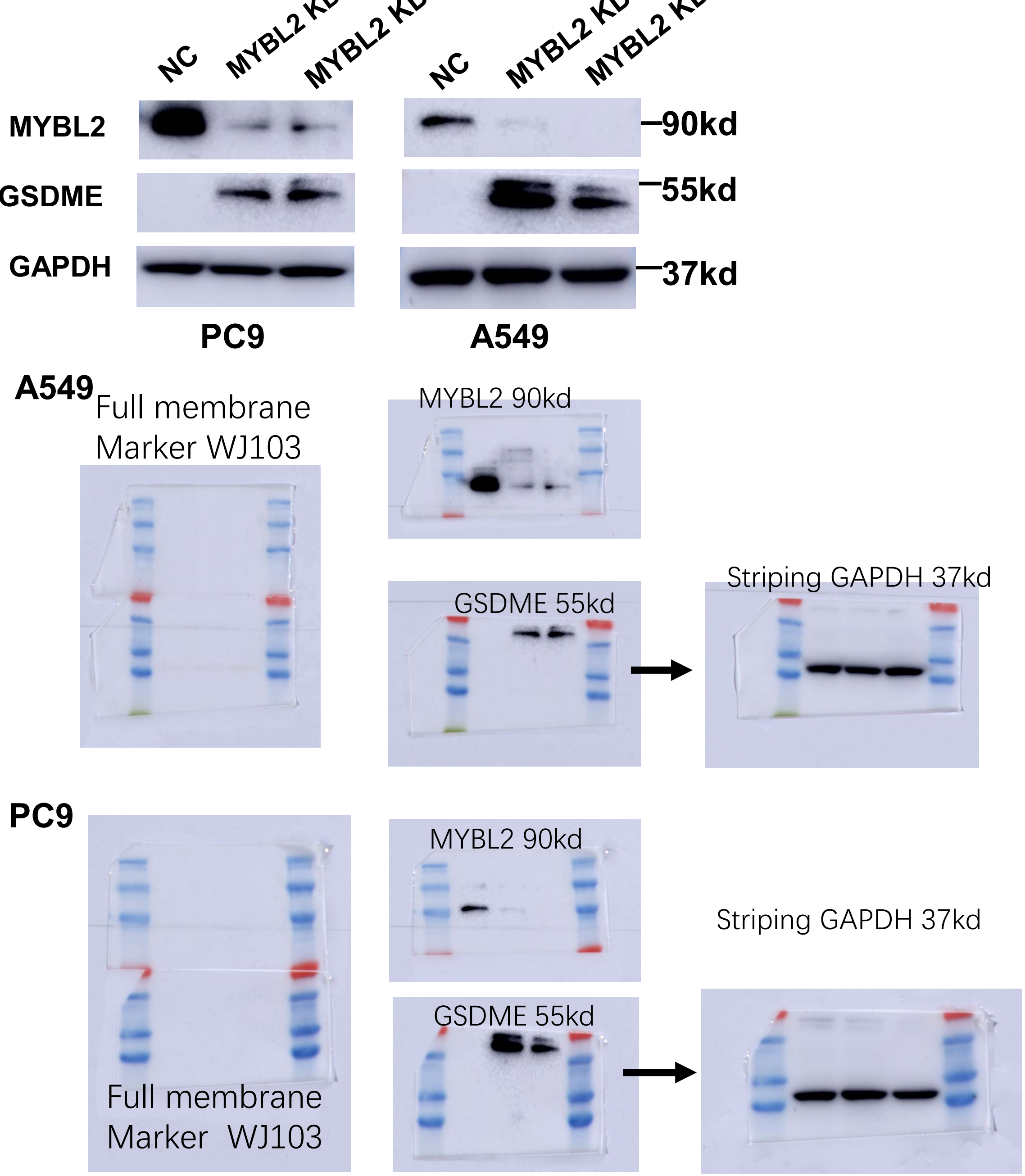

Figure 4D

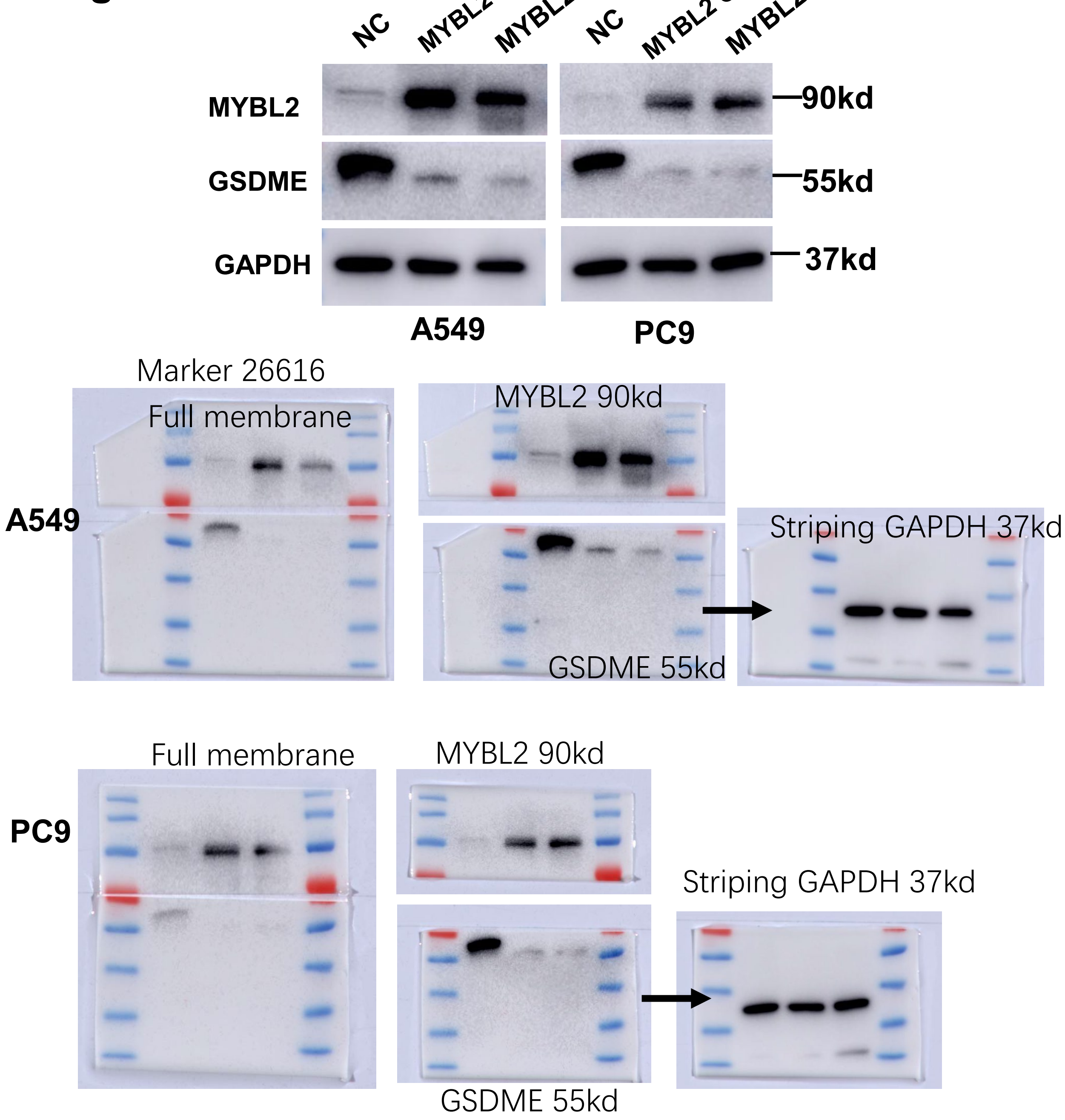

Figure 5C-D

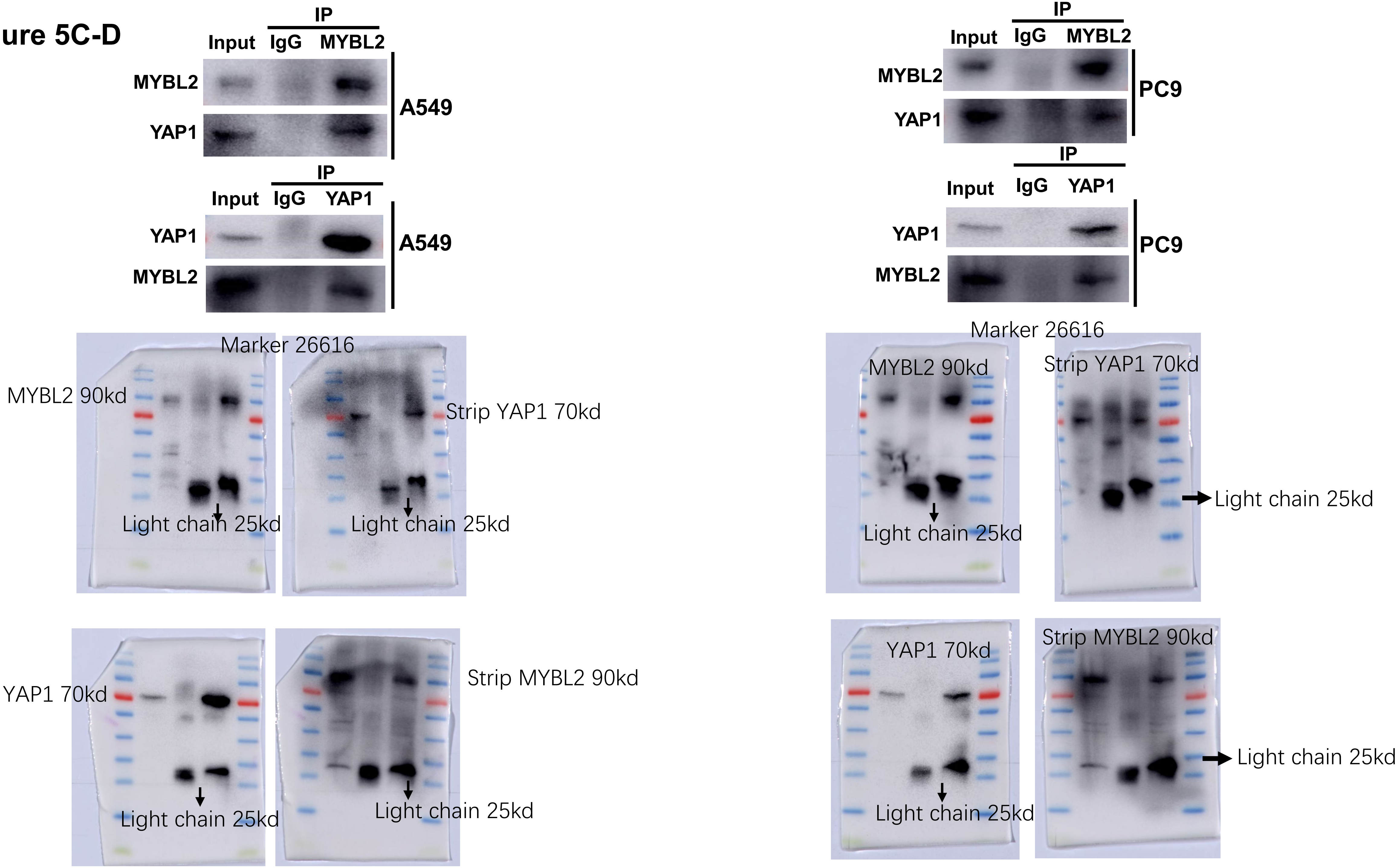
IP
Input
IgG
MYBL2
YAP1
A549
PC9
Marker 26616
MYBL2 90kd
Strip YAP1 70kd
YAP1 70kd
Strip MYBL2 90kd
Light chain 25kd

Figure 5E-F

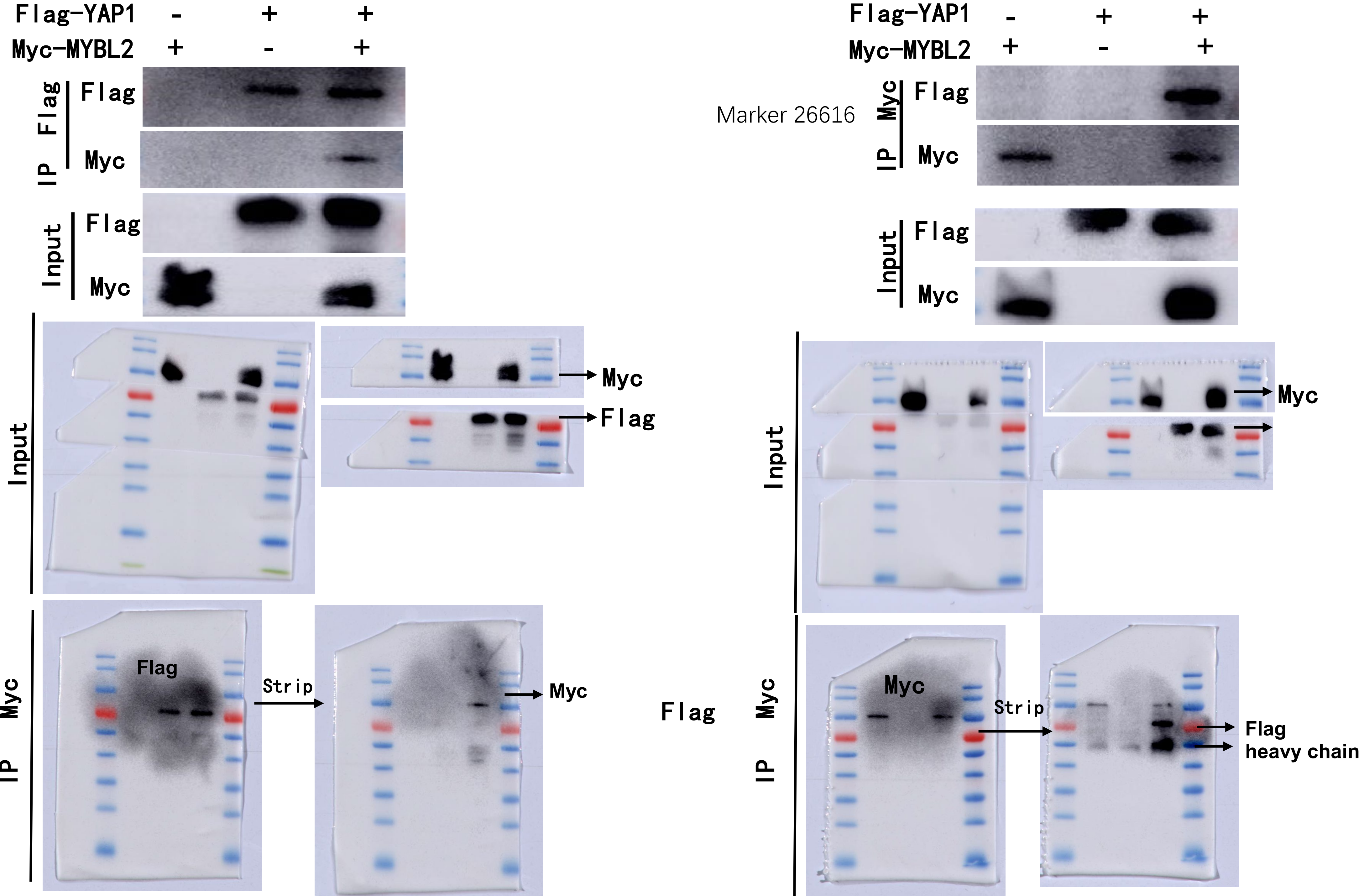
Flag-YAP1 - + +
Myc-MYBL2 + - +
Marker 26616
IP Flag
Flag
Myc
Input
Flag
Myc
Input
Myc
Flag
IP Myc
Flag
Strip
Myc
Flag-YAP1 - + +
Myc-MYBL2 + - +
Marker 26616
IP Myc
Flag
Myc
Input
Flag
Myc
Input
Myc
Flag
IP Flag
Myc
Strip
Flag
heavy chain

Figure 5E

Marker 26616

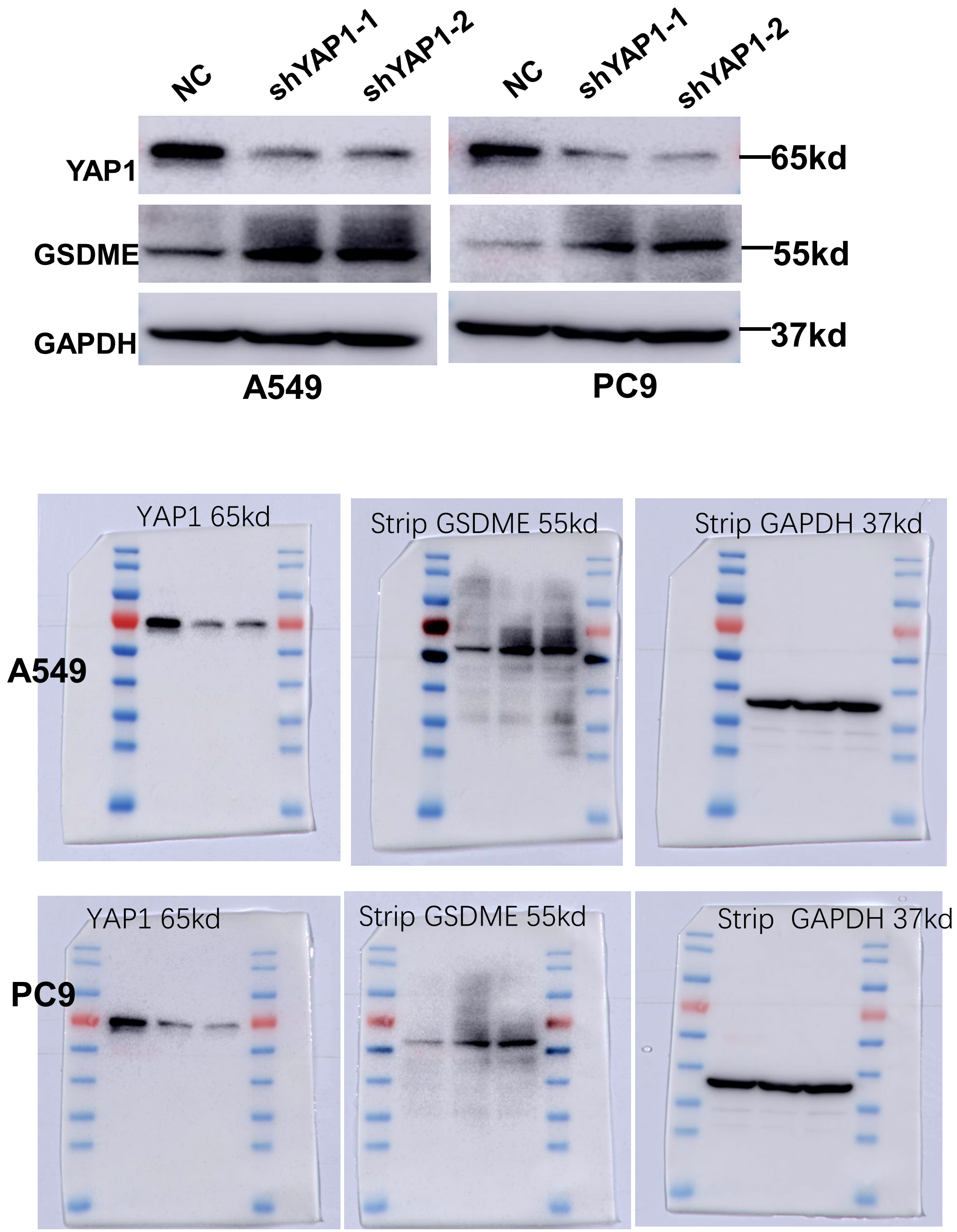

Figure 5F

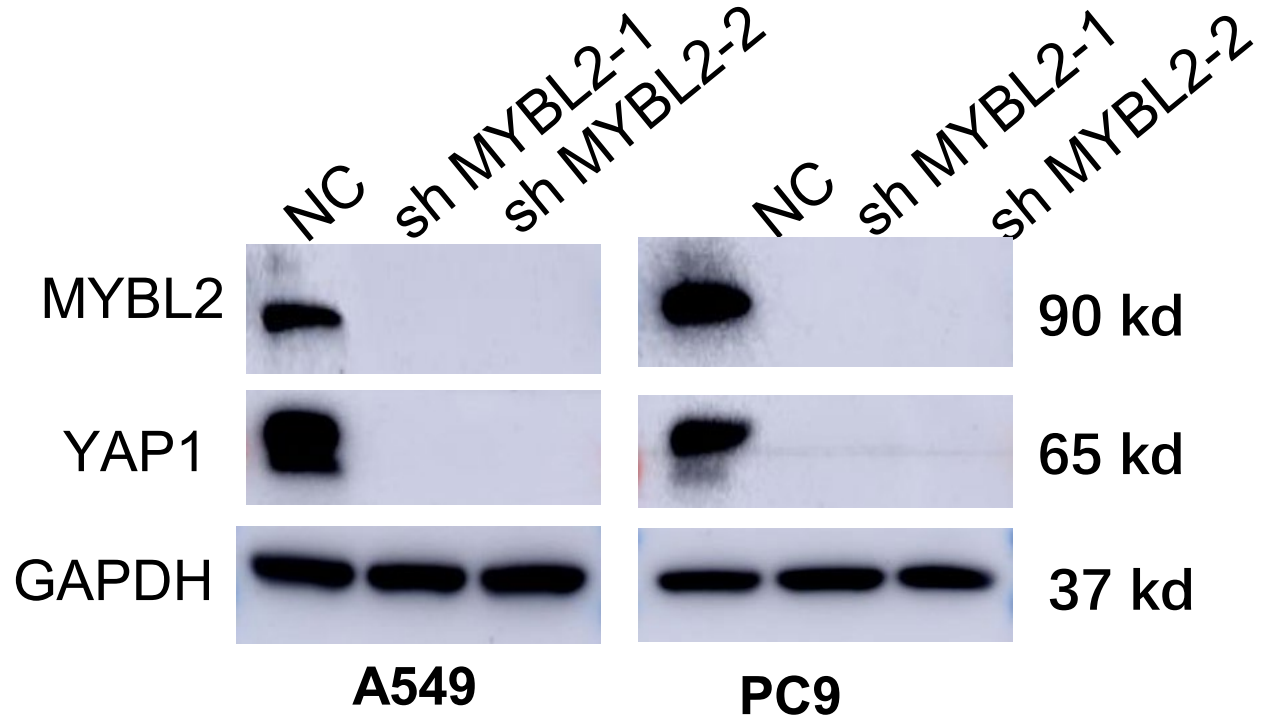

A549

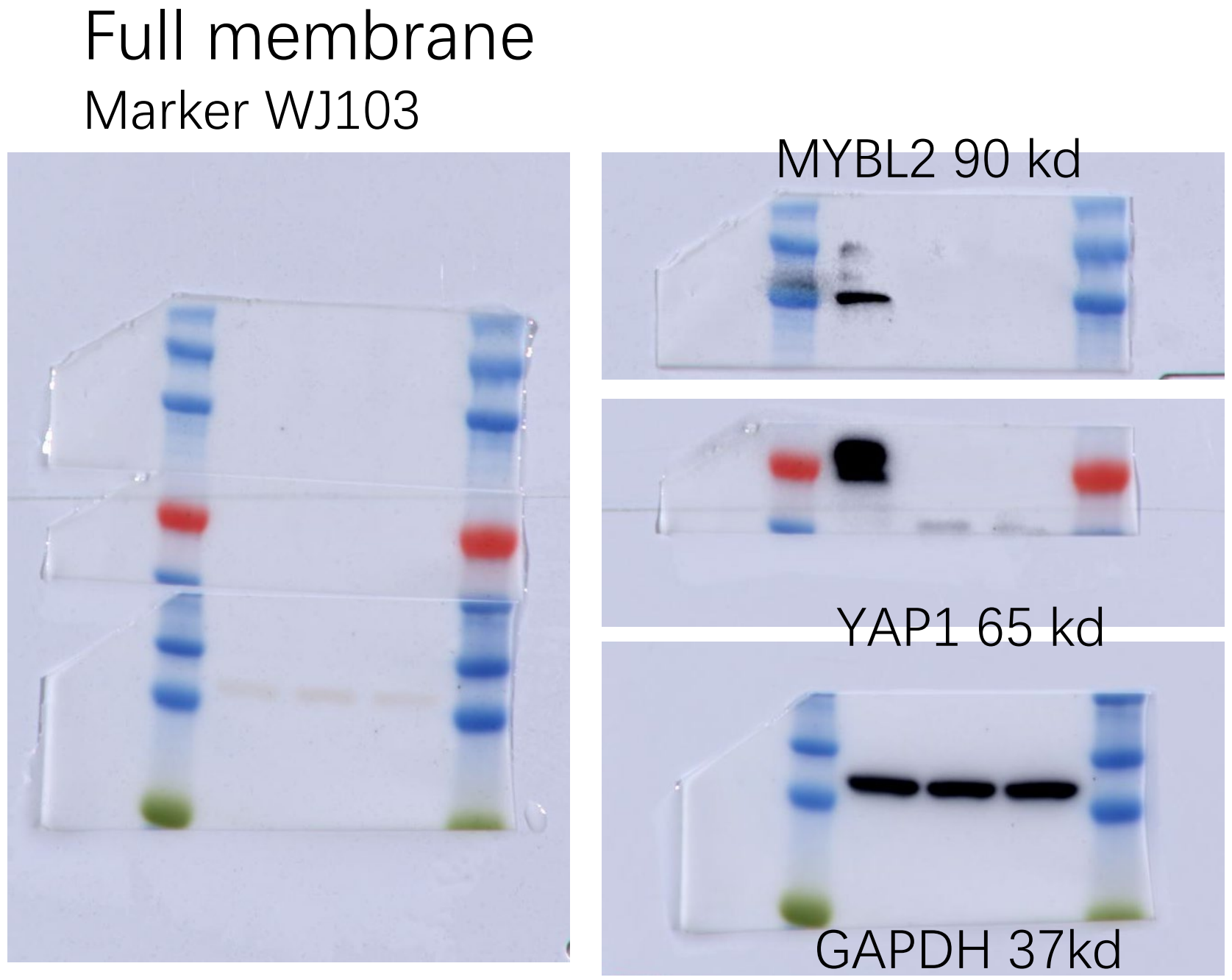

PC9

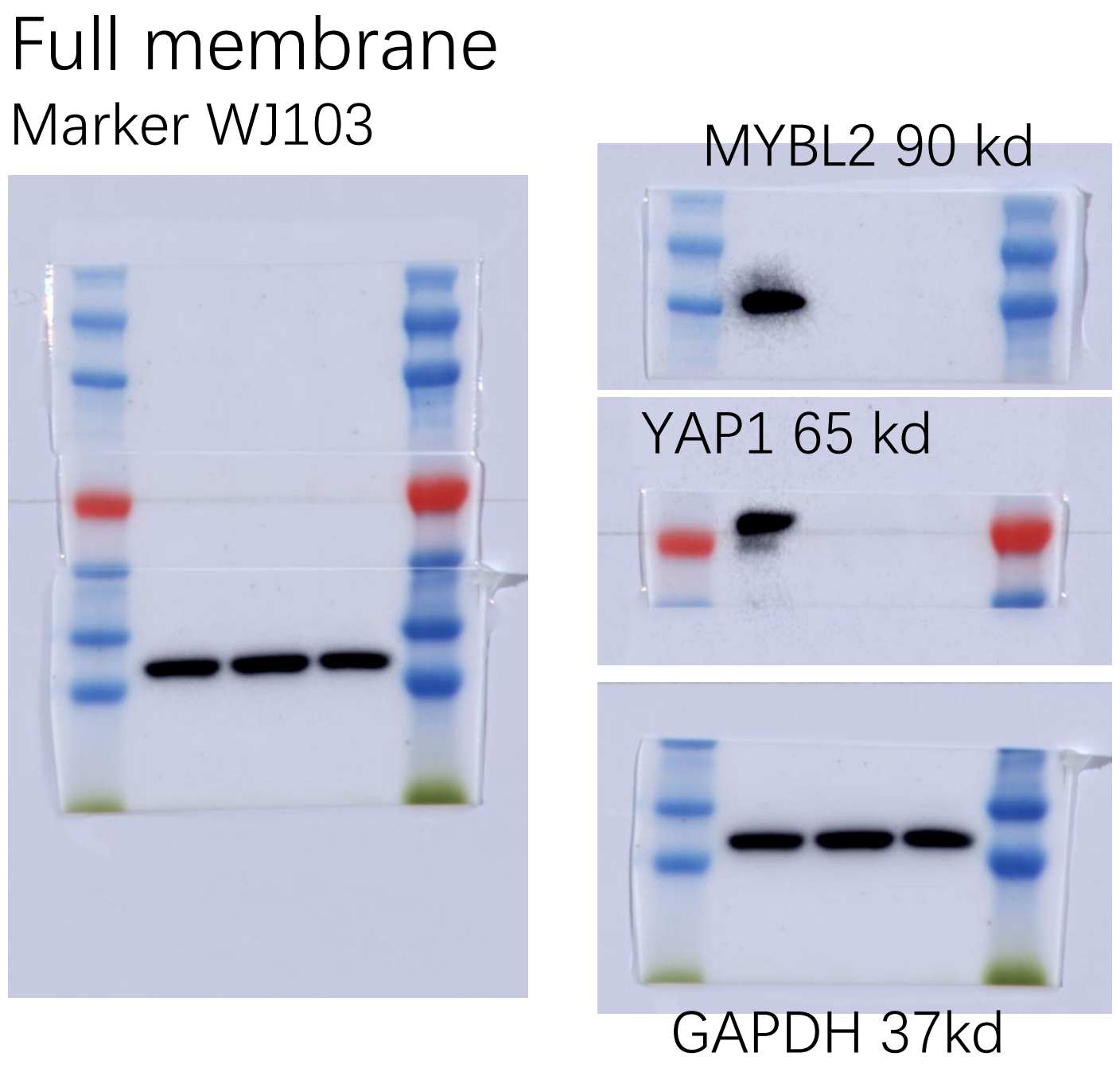

Figure 5N Marker 26616

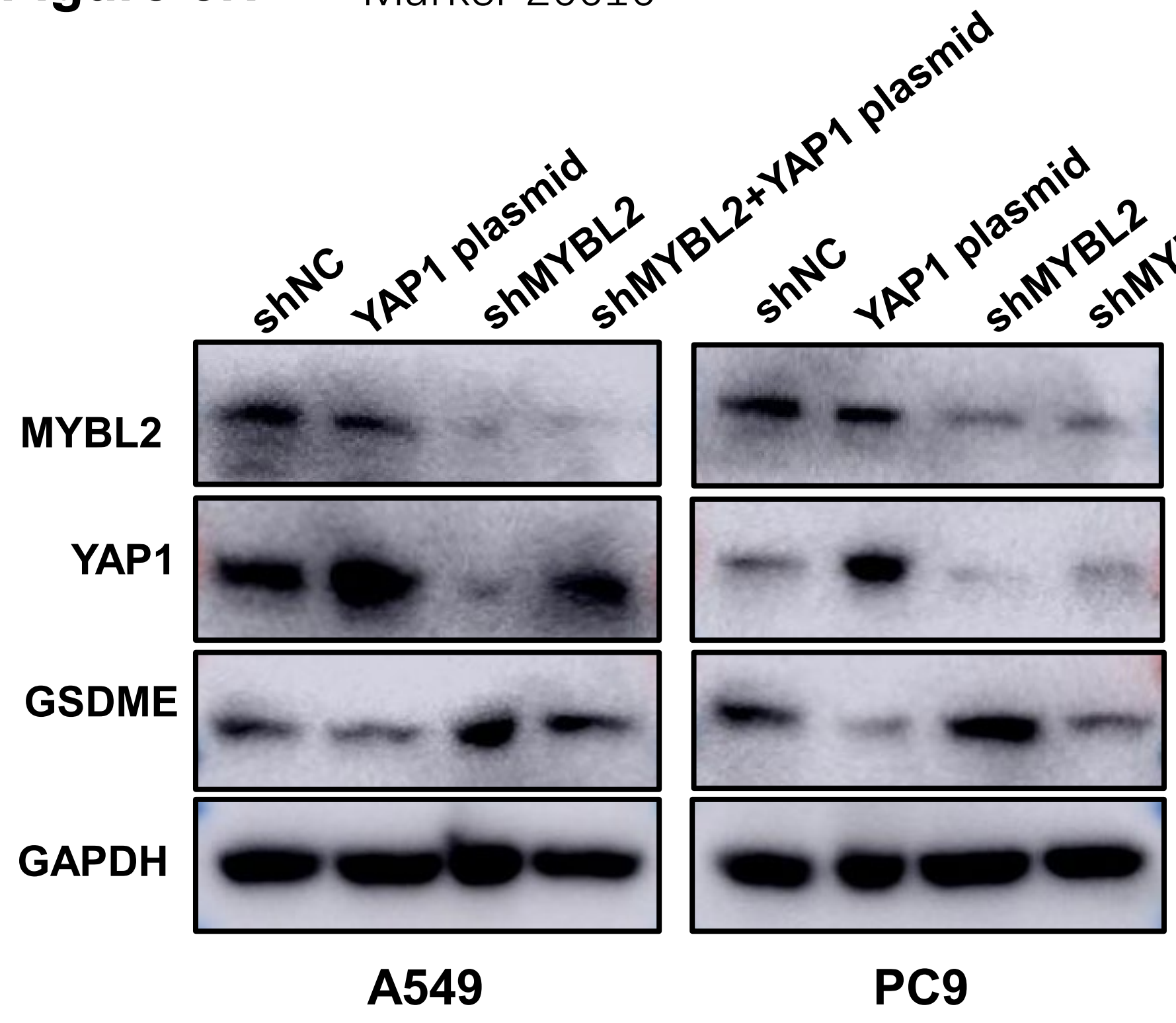

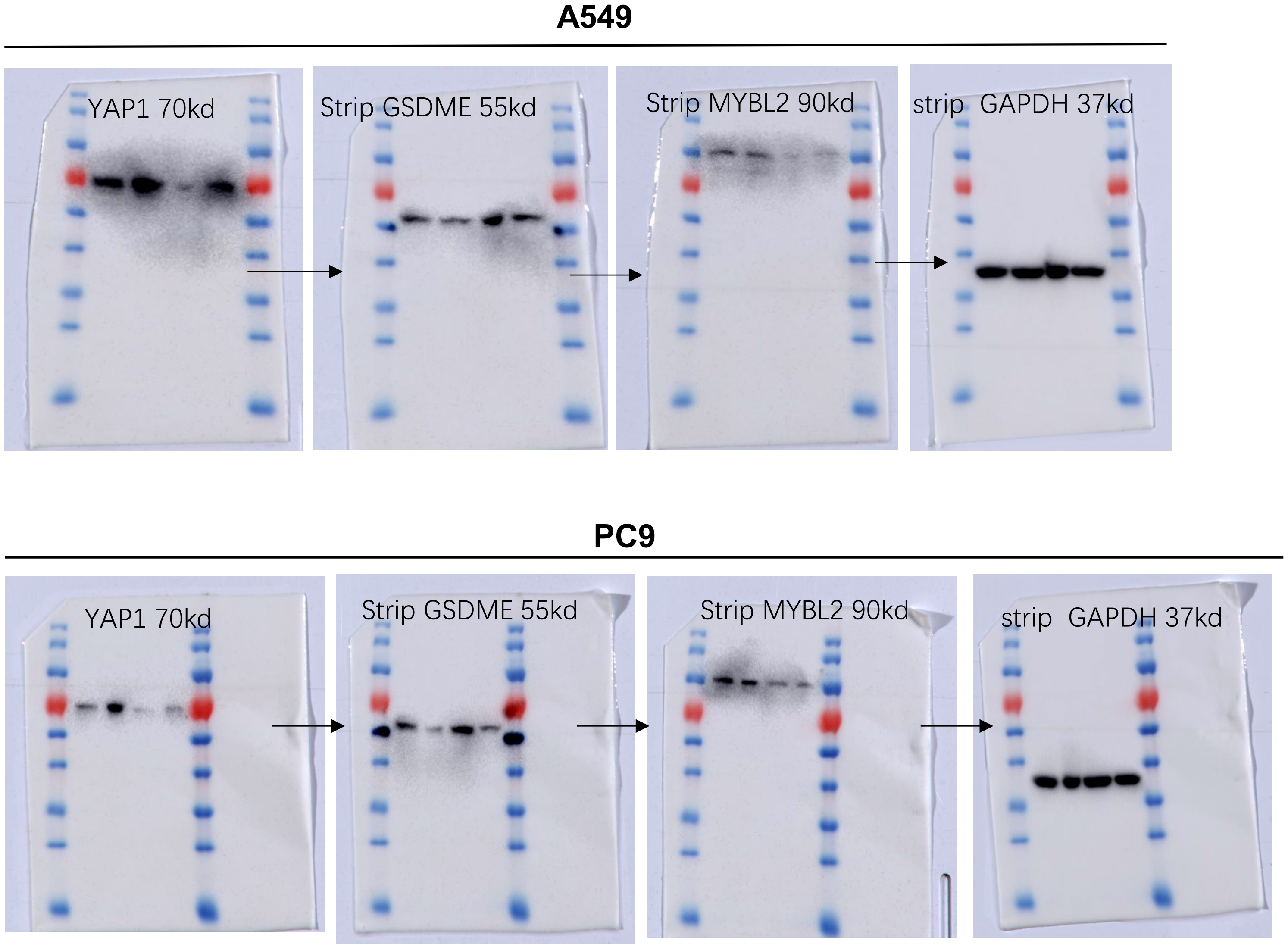

Supplementary Figure 3E

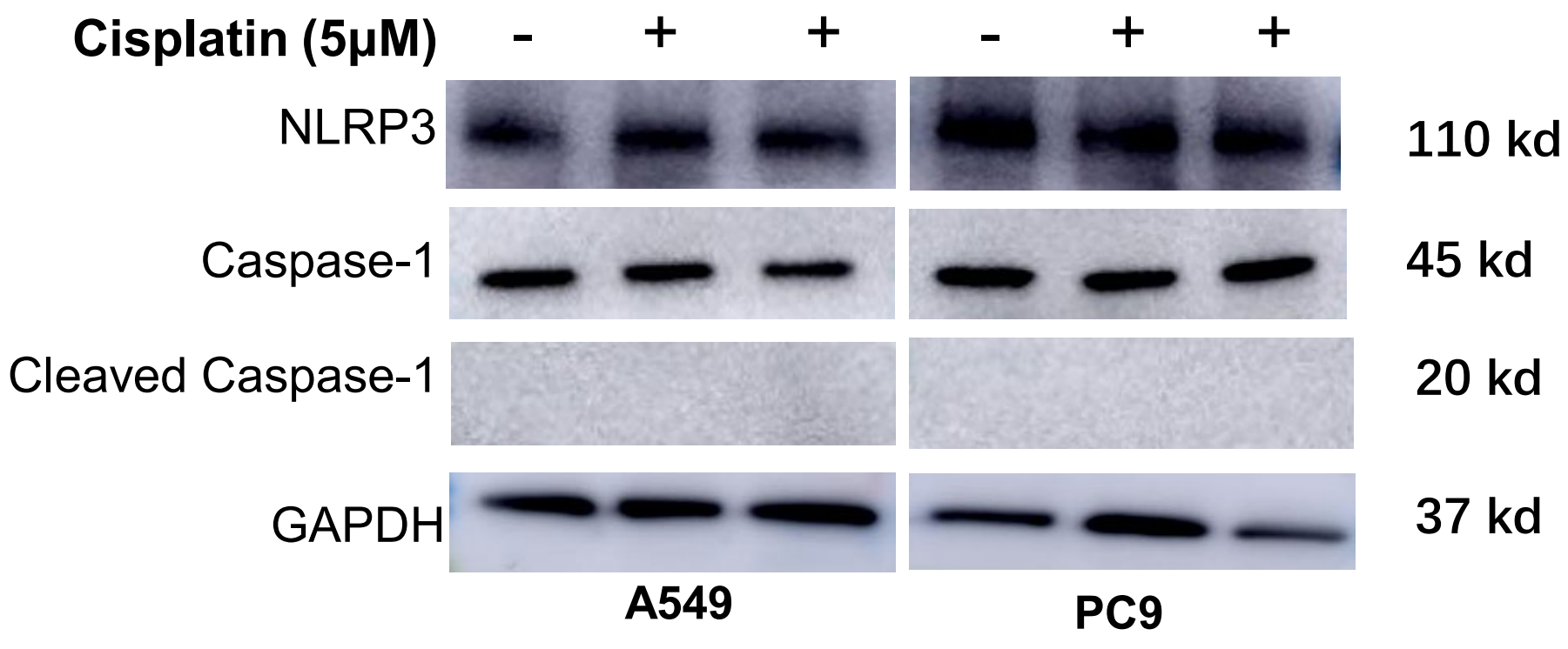

Full membrane Marker WJ103

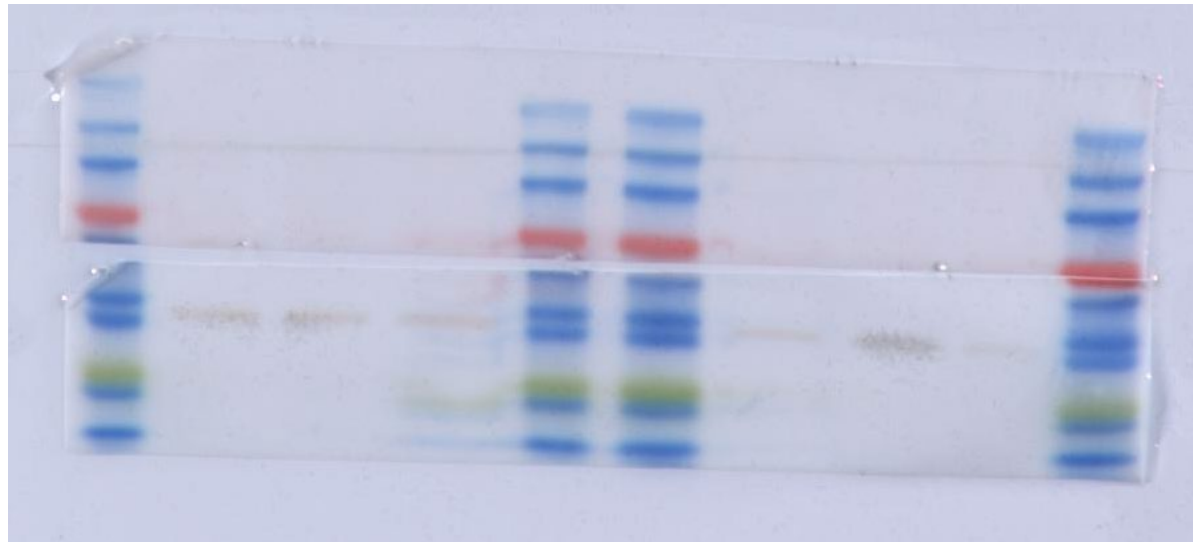

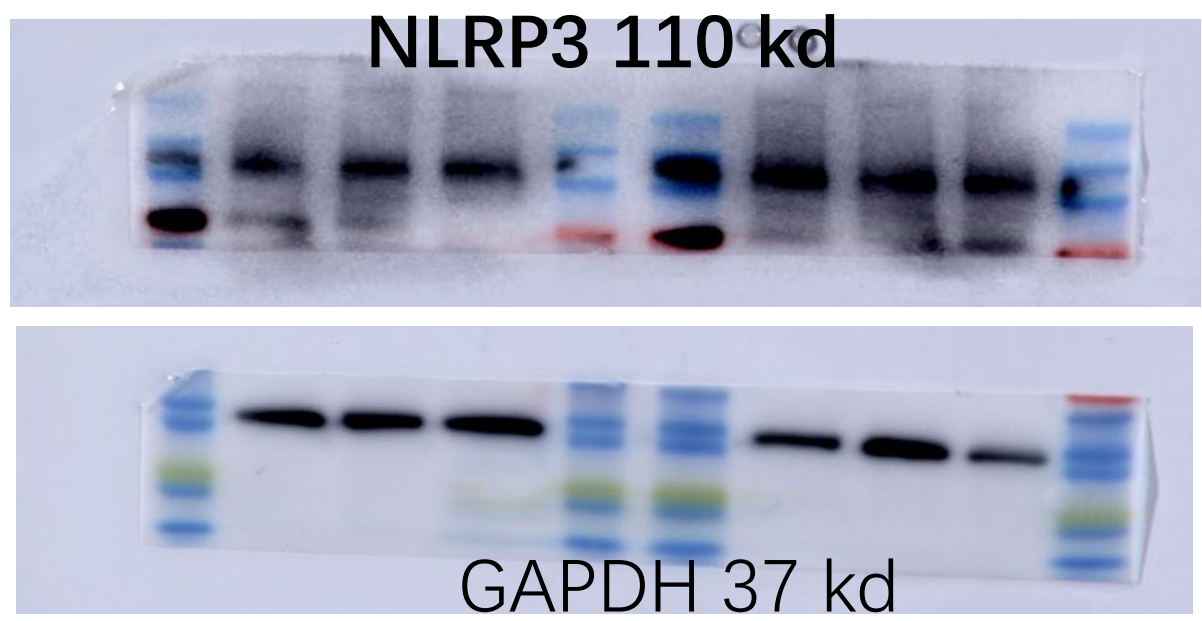

Experimental condition:
Electrophoretic conditions: 10% agar gel, 120min
Film transfer conditions: constant electric current 300mA, 75min

Full membrane Marker WJ103

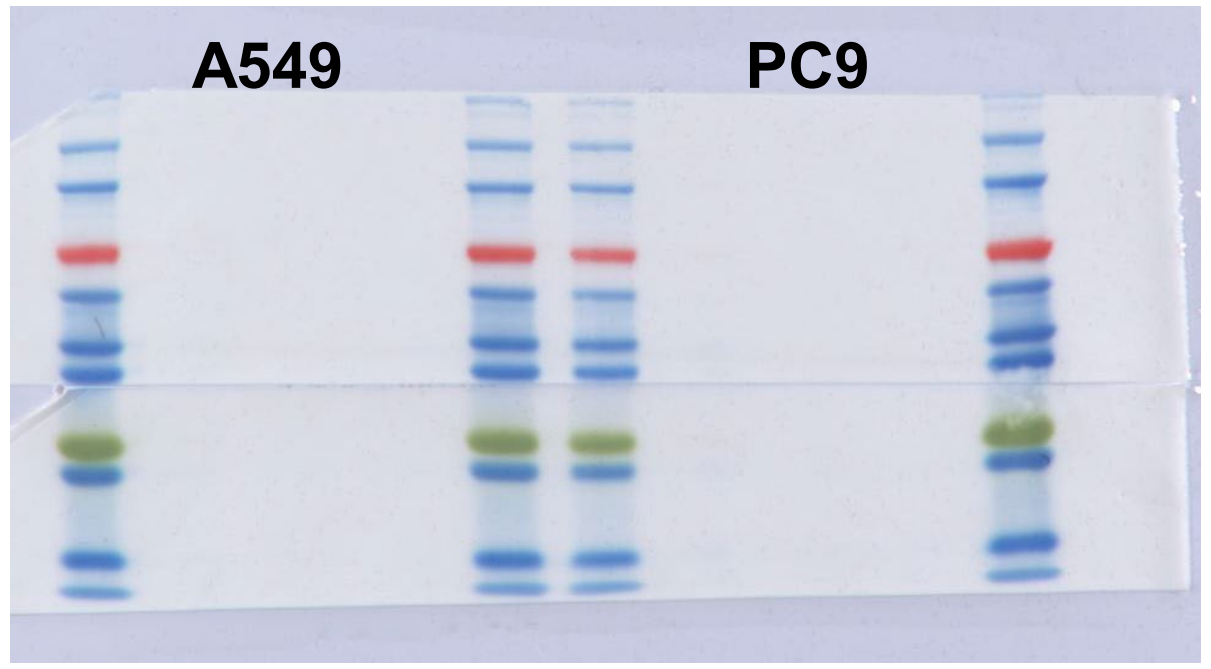

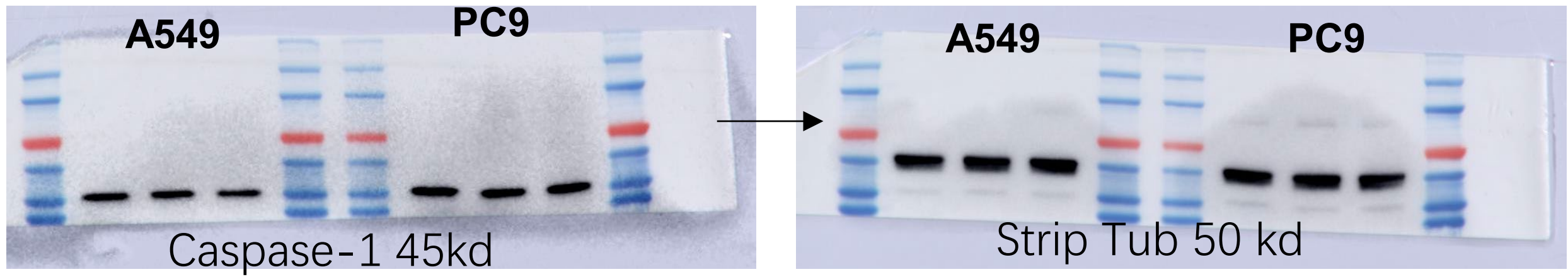

We used tubulin (50kd) as an internal reference protein to avoid overlapping with the caspase-1 band(42kd).

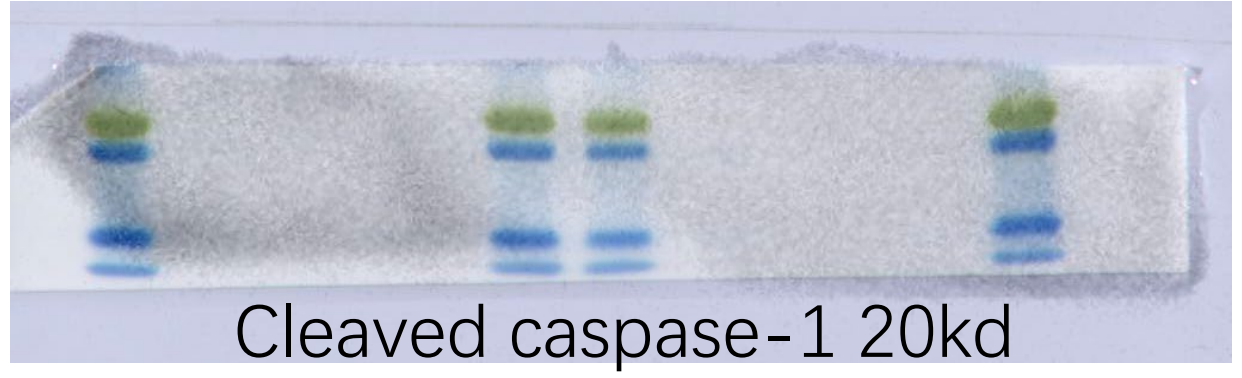

Experimental condition:
Electrophoretic conditions: 12.5% agar gel, 60min
Film transfer conditions: constant electric current 300mA, 60min
