# Supplementary material for: MYBL2 impedes cisplatin sensitivity through suppressing GSDME-mediated pyroptosis in lung adenocarcinoma
Source: Cell Death Discov. 2026 Jun 2;12:327. doi: 10.1038/s41420-026-03175-y (PMC13443601; doi:10.1038/s41420-026-03175-y)
Supplement: Supplementary file 4 [file 41420_2026_3175_MOESM5_ESM.docx]

**Table 1**

| Primers | Primer sequences (5′ to 3′) |
| --- | --- |
| GSDME-Forward primers | CCAGTTTTTATCCCTCACCCTTG |
| GSDME-Reverse primers | CAAACTTGCCCTCGTATTTCACA |
| GSDMD-Forward primers | GCTGGTTATTGACTCTGACTTGGAC |
| GSDMD-Reverse primers | GACCCCATCTGTCAGGAAGTT |
| MYBL2-Forward primers | CTTGAGCGAGTCCAAAGACTG |
| MYBL2-Reverse primers | AGTTGGTCAGAAGACTTCCCT |
| YAP1-Forward primers | TAGCCCTGCGTAGCCAGTTA |
| YAP 1-Reverse primers | TCATGCTTAGTCCACTGTCTGT |
| ACTIN-Forward primers | TGGAGAAAATCTGGCACCACACC |
| ACTIN-Reverse primers | GATGGGCACAGTGTGGGTGACCC |
